# The enigmatic HCN channels: A cellular neurophysiology perspective

**DOI:** 10.1002/prot.26643

**Published:** 2023-11-19

**Authors:** Poonam Mishra, Rishikesh Narayanan

**Affiliations:** 1 Department of Neuroscience, Yale School of Medicine, Yale University, New Haven, Connecticut, USA; 2 Cellular Neurophysiology Laboratory, Molecular Biophysics Unit, Indian Institute of Science, Bangalore, India

**Keywords:** homeostasis, impedance, inductance, intrinsic excitability, intrinsic plasticity, oscillations, resonance, temporal summation

## Abstract

What physiological role does a slow hyperpolarization-activated ion channel with mixed cation selectivity play in the fast world of neuronal action potentials that are driven by depolarization? That puzzling question has piqued the curiosity of physiology enthusiasts about the hyperpolarization-activated cyclic nucleotide-gated (HCN) channels, which are widely expressed across the body and especially in neurons. In this review, we emphasize the need to assess HCN channels from the perspective of how they respond to time-varying signals, while also accounting for their interactions with other co-expressing channels and receptors. First, we illustrate how the unique structural and functional characteristics of HCN channels allow them to mediate a slow negative feedback loop in the neurons that they express in. We present the several physiological implications of this negative feedback loop to neuronal response characteristics including neuronal gain, voltage sag and rebound, temporal summation, membrane potential resonance, inductive phase lead, spike triggered average, and coincidence detection. Next, we argue that the overall impact of HCN channels on neuronal physiology critically relies on their interactions with other co-expressing channels and receptors. Interactions with other channels allow HCN channels to mediate intrinsic oscillations, earning them the “pacemaker channel” moniker, and to regulate spike frequency adaptation, plateau potentials, neurotransmitter release from presynaptic terminals, and spike initiation at the axonal initial segment. We also explore the impact of spatially non-homogeneous subcellular distributions of HCN channels in different neuronal subtypes and their interactions with other channels and receptors. Finally, we discuss how plasticity in HCN channels is widely prevalent and can mediate different encoding, homeostatic, and neuroprotective functions in a neuron. In summary, we argue that HCN channels form an important class of channels that mediate a diversity of neuronal functions owing to their unique gating kinetics that made them a puzzle in the first place.

## 1 Introduction

**Figure 1:**
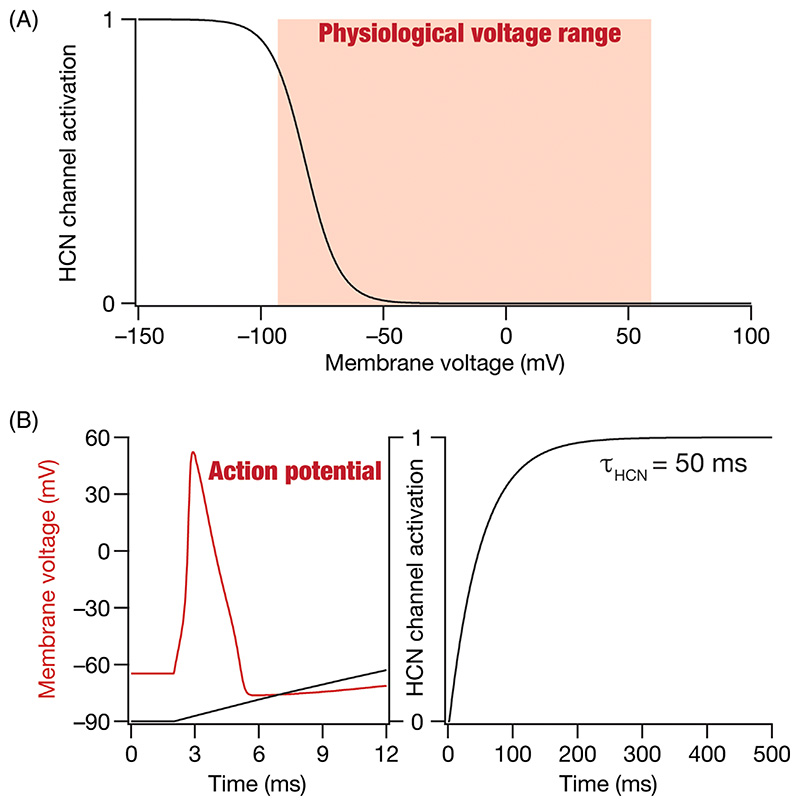
The unique activation profile of HCN channels. (A) The typical physiological range of neuronal membrane voltages is between −80 mV and +60 mV. HCN channels, however, have a uniquely hyperpolarization-activated profile. The half-maximal activation voltage of these channels is around −80 mV with channels closing at typical supra-threshold voltages (>−45 mV). (B) Action potentials are fast events that last for around a millisecond period. HCN channel activation (obtained with a large hyperpolarizing voltage pulse) is slow, with time constants on the order of tens to thousands of milliseconds (shown is an example with activation time constant, τ_HCN_ = 50 ms). Shown is the overlay of HCN-channel activation on the time course of action potential to demonstrate the slow nature of HCN channels. Note that the overlay is solely intended for conveying the differences in time courses of HCN channel activation (slow) and an action potential (fast). The two plots representing different quantities derived from distinct experimental conditions have no other relationship. The voltage dependent properties and activation time constants of HCN channels are representative of typical HCN channels and are derived from electrophysiological recordings.^15,18,37^

Ion channels are membrane proteins that mediate most electrical activity in excitable cells, including neurons. The physiological range of neuronal membrane voltages fall famously between the equilibrium potential for potassium (around −80 mV) and the equilibrium potential for sodium (around +60 mV). So, what function does a membrane protein that reaches only about half its maximal activation at hyperpolarized voltages (around −80 mV) achieve, especially when the activation of the channel continues to reduce as the voltage depolarizes towards +60 mV (Figure 1A)? Further, in the fast-paced setting of neuronal action potentials that last about a millisecond (Figure 1B), what function can this membrane protein with slow kinetics (on the order of 10–1000 s of milliseconds; Figure 1B) even achieve? Why would such a protein allow both sodium and potassium ions to pass through? These puzzling questions pertain to the hyperpolarization-activated cyclic-nucleotide-gated (HCN) channels that occupy a rather unique place with their slow kinetics and voltage-dependent activation profiles dominant at hyperpolarized voltages. This unique class of voltage-gated ion channels is made of four different main subunits (HCN1–HCN4), with several different auxiliary subunits. Ionic currents mediated by these channels have been known by different names^1–4^: the pacemaker current, the funny current (*I_f_*), the queer current (*I_q_*), and the *h* current (*I_h_*).

In this review, we provide a synthesis of the implications for the expression of HCN channels in neurons and their compartments. We argue that the puzzling biophysical characteristics of HCN channels are the very reason for their ability to confer unique physiological capabilities upon neurons that they express in. Over the decades, the confounding biophysical characteristics have posed several questions and have been a source of numerous fundamentally important debates about the relationship between ion-channel properties and neuronal function. We provide an overview of some of the important questions and debates about HCN channels and present a perspective that synthesizes different viewpoints. We particularly emphasize the *interactions* viewpoint, where the implications of ion channels on neuronal physiology ought to be viewed in the context of the *multifarious interactions* of these channels with morphological, passive, and active properties of neurons. We argue that HCN channels confer upon neurons a diversity of unique physiological capabilities in the context of these interactions. The diversity of conferred physiological capabilities is further enhanced when the ability of these ion channels to change in response to activity patterns is accounted for.

Whereas our review focuses on the unique physiological capabilities that the HCN channel confers upon single neurons, there are several recent reviews that focus on different aspects of HCN channel structure and function.^1,5–12^

## 2 HCN Proteins: Structure and its Relationship to their Unique Channel Physiology

The structure of voltage-gated ion channels allows them to detect changes in the transmembrane potential and effectuate conformational changes in the protein structure towards allowing flow of specific ions. Unlike most voltage-gated ion channels that increase their conductivity in response to *depolarization*, HCN channels allow flow of ions across the membrane in response to *hyperpolarization* of the membrane potential. Further, HCN channels are non-specific monovalent cationic channels that allow sodium and potassium ions (permeability ratio for Na^+^:K^+^ is ˜1:4), setting their reversal potential (the voltage at which the current reverses its direction) to be unique at around −20 to −30 mV. Whereas the voltage-dependent conformational changes mediate the gating properties of ion channels, their selectivity to specific ions is typically conferred by selectivity filters in their pore region. What structural differences in the protein molecule, both in terms of voltage-dependent conformational changes as well as the design of the selectivity filter, bestow these unique characteristics upon HCN channels?

The HCN channel is a tetrameric structure made of the pore-forming HCN1–HCN4 subunits in mammals.^13–17^ Functionally, these subunits majorly differ in terms of their voltage-dependent gating profiles, activation kinetics, and their dependence on cyclic nucleotides. Specifically, homo-tetramers made of HCN1 subunits yield the fastest HCN channels with the activation time constant in the 30–300 ms range.^15,18^ On the other end, homo-tetramers made of HCN4 sub-units yield the slowest of the HCN channels with an activation time constant on the order of few hundred milliseconds to a few seconds.^14,16,19^ In addition, HCN2 and HCN4 channels are extremely sensitive to binding of intracellular cyclic adenosine monophosphate (cAMP), driving a large depolarizing shift to the voltage-dependent activation profile.^14,16,19–21^ On the other hand, HCN1 and HCN3 channels show weak sensitivity to cAMP binding.^20–23^ As HCN channels can be homo- or hetero-tetrameric, the physiological characteristics of native HCN channels and their modulation by cAMP are dependent on the co-expression profiles and stoichiometry associated with the different pore-forming subunits^20,24,25^ and other auxiliary subunits.^2,26^ The expression profiles of these subunits are variable across different cell types of the brain.^18,27^ As this review primarily focuses on the functions of HCN channels in cortical and hippocampal pyramidal neurons, it is essential to note that these cells predominantly express HCN1 and HCN2.^18,27^

Each pore-forming HCN-channel subunit is comprised of six transmembrane segments S1–S6. The S4 segment acts as a voltage sensor as it is comprised of multiple repeats of charged residues. The unique reverse voltage dependence of HCN channels emerges as a consequence of the absence of the domain swapping of voltage sensors as well as the specific hyperpolarization-induced conformational changes associated with the S4 helix. Another important characteristic of HCN channels, their ability to conduct both sodium as well as potassium, is thought to be a consequence of the differences in the selectivity filter compared to potassium channels. Structurally, the HCN-channel selectivity filter is very similar to the potassium-channel selectivity filter. Strong selectivity for potassium ions in potassium-channel selectivity filter (permeability ratio for Na^+^:K^+^ is ˜1:1000) manifests due to the presence of four ion-binding sites in the selectivity filter. In contrast, HCN channels preserve only two of these potassium ion-binding sites with the outer half of the selectivity filter dilated. These structural properties associated with the voltage-sensor conformational changes and the selectivity filter confer the unique physiological characteristics of HCN channel as a hyperpolarization-activated, non-specific monovalent cation channels.^9,17,18,28–32^

## 3 The Resting Viewpoint: Conductance-Current Balance and Input Resistance

The resting membrane potential (RMP) of a neuron is defined as the steady-state membrane potential of a neuron at zero current injection. The RMP of a neuron is governed by the concentration gradients associated with the different ions and the membrane permeability for each of these different ions under resting conditions.^33–35^ As resting membrane permeability is predominantly mediated by potassium leak channels, RMP stays closer to the equilibrium potential of potassium (around −70 mV). However, in neurons that express HCN channels, a mixed cation channel that is permeable to both potassium and sodium ions and is open under resting conditions (Figure 1A), there is a strong inward current mediated by sodium ions. Thus, RMP must account for enhanced sodium permeability mediated by HCN channels, which results in a *depolarization of RMP* in the presence of HCN channels.^36–38^

Steady-state input resistance (*R*_in_) is a well-established measure of neuronal gain and excitability. An important contributor to *R*_in_ is the set of ion channels that are in open state at the voltage where *R*_in_ is measured. With each open ion-channel acting as a parallel conductance, the overall conductance increases when there are more ion channels present on the membrane. As resistance is inversely related to conductance, a higher number of conducting ion channels (at the voltage-level where the measurement is being carried out) results in a reduction of the steady-state gain, *R*_in_. With reference to the expression of HCN channels, with their voltage-dependent profile implying that there are open HCN channels at rest, these observations translate to a reduced *R*_in_ in the presence of HCN channels.^36–38^ Thus, the expression of HCN channels enhances the overall resting conductance and *reduces the excitability* of a neuron.

Together, the resting *current* through HCN channels *depolarizes* the membrane which takes the neuron closer to action potential threshold, thereby making it easier for an incoming current to elicit an action potential. This implies an enhancement in excitability owing to HCN-channel expression. In striking contrast, the resting *conductance* consequent to HCN-channel expression is higher, which reduces *R*_in_, implying a reduction in the voltage response to incoming current. This implies a reduction in excitability owing to HCN-channel expression. The conductance and the current mediated by resting HCN channels, therefore, act in opposite directions in terms of how they regulate excitability. This dichotomy between the current- vs. conductance-based viewpoints *under resting conditions* has been a source of considerable debate about the impact of HCN channels on neuronal excitability.^2,3,26,30,36–54^

Much of the debate has centered around the question of how HCN channels, which mediate an *inward current*, could *reduce* the excitability of neurons. Here, we argue that the conductance-current conundrum is simply a reflection of looking at HCN channels from the resting state of a neuron. If one considers the *dynamic viewpoint* (as to how HCN channels respond to incoming time-varying stimuli) and assess whether the inward current is enhanced or shut down by a depolarizing input, the resolution to *how* an inward current could reduce excitability becomes clear. The unique characteristics of the HCN channels mediating a *hyperpolarization-activated inward current* ensure that under a dynamic perspective, the ability of HCN channels to reduce excitability is obvious without an element of doubt. In addition, and importantly, we will also address the conductance-current conundrum from the perspective of interactions with other ion channels that co-express with HCN channels.

In what follows, we present the dynamic viewpoint that views HCN channels as a *negative feedback loop* that mediates a reduction in excitability, and an interactions viewpoint which emphasizes the need to assess HCN-channel physiology *in the context of the global structure* involving the expression of other channels.

## 4 Dynamic Viewpoint: Negative Feedback Loop

Neuronal excitability is not defined by merely the RMP and *R*_in_ of a neuron. These measures are steady-state measures of neuronal physiology and do not account for neural responses to time-varying inputs under typical *in vivo* conditions. In addition, *R*_in_ is not governed merely by the resting conductance associated with the ion channels that are expressed there, but also with the *dynamics* of how these channel conductances dynamically change with input stimuli.

**Figure 2:**
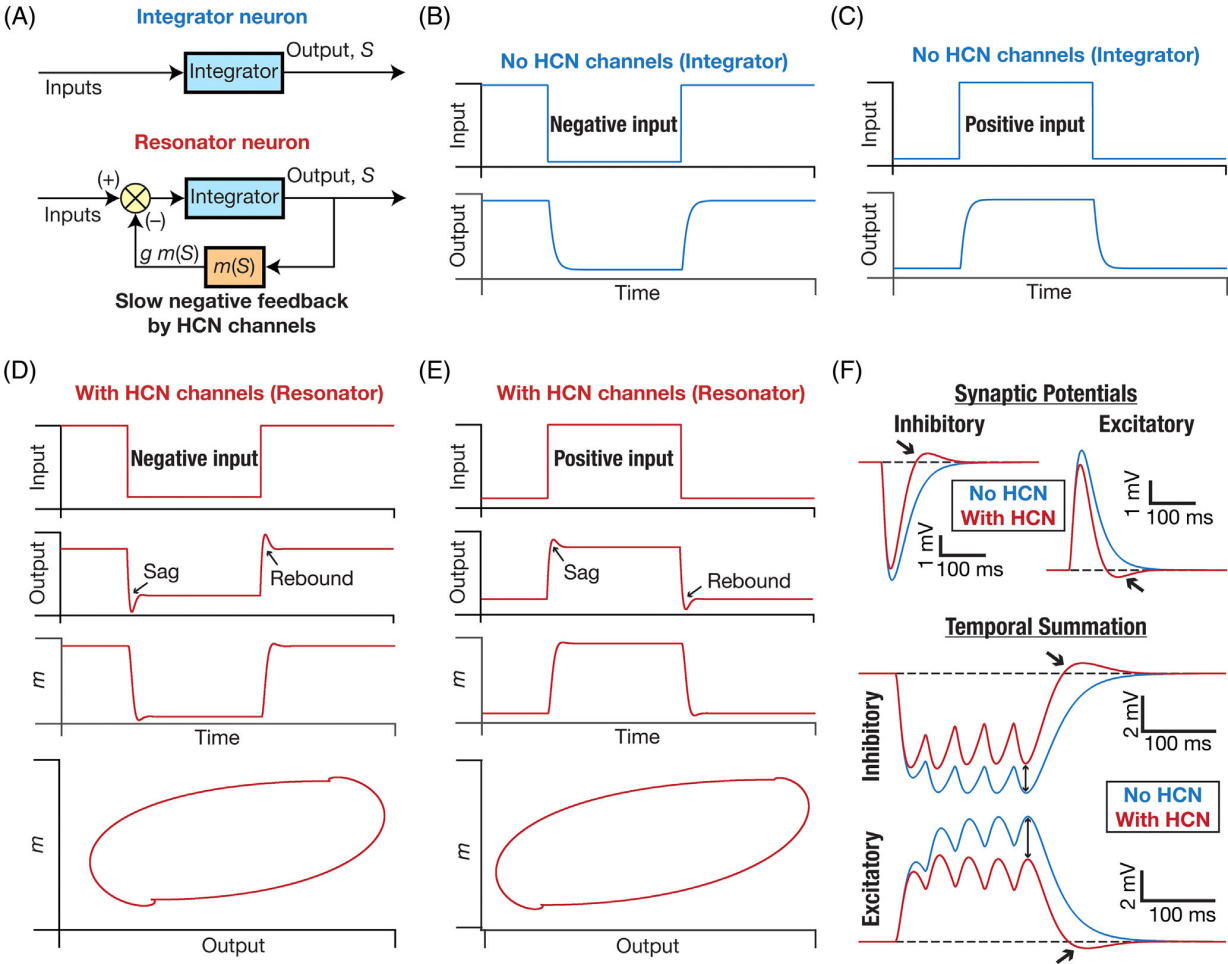
The presence of HCN channels introduces a slow negative feedback loop that mediates sag/rebound and suppresses temporal summation in neurons. (A) *Top*, The lipid bilayer on the plasma membrane along with the ion channels there introduce an integrator-like structure. The presence of HCN channels introduces a slow negative feedback loop through a current that is dependent on the output voltage. The hyperpolarization-activated nature of the inward HCN current translates to a negative feedback structure, with the slow kinetics of HCN channels mediating the slow feedback.^11,40,89,147,256^ (B, C) Temporal evolution of the output (*S*) of an integrator neuron in response to negative (B) or positive (C) inputs. (D, E) Temporal evolution of the output (*S*) of a resonator neuron and the state variable related to the negative feedback (*m*) in response to negative (D) or positive (E) inputs. The bottom-most plots constitute a phase-plane representation (of *m* vs. the output *S*) of the dynamics depicted in the respective panels. Note the manifestation of sag and rebound in the output traces. The manifestation of sag and rebound in the presence of HCN channels has been electrophysiologically observed in several neuronal subtypes.^256^ (F) *Top*, Impact of the presence of HCN channels on single inhibitory (left) or excitatory (right) synaptic potentials. Note the small reduction in amplitude, a pronounced change in decay kinetics, and the rebound in synaptic potentials obtained in the presence of HCN channels. *Bottom*, Impact of the presence of HCN channels on temporal summation of multiple inhibitory or excitatory synaptic potentials. Note the large reduction in temporal summation (compare amplitudes of the last synaptic potential without and with HCN; two-sided arrows), a pronounced change in decay kinetics, and the rebound (single-sided arrows) in synaptic potentials obtained in the presence of HCN channels. The strong impact of HCN channels on temporal summation has been electrophysiologically demonstrated in multiple neuronal subtypes.^37,55,56,129^ Parts of panels A–C in this figure have been modified from Mittal and Narayanan.^89^

Let us consider an example. Consider a neuron that does not express HCN channels (Figure 2A). The response of such a neuron to a negative or positive pulse current is typically an exponential-like charging curve that settles at a hyperpolarized or depolarized steady-state voltage value, respectively (Figure 2B,C). Now, let’s add HCN channels to this passive neuron that acts as an *integrator* of incoming information. The HCN channel mediates a *voltage-dependent* conductance. Therefore, as the neuron responds to a positive current input with a depolarizing response, the resulting voltage deflection *reduces* the HCN-channel conductance (Figure 1A). A reduced channel conductance in turn *reduces* the inward current that is mediated by the HCN channel, which results in *hyperpolarization* of the neuron. Thus, depolarization of a neuron by an input stimulus results in a hyperpolarization of the membrane potential because the HCN channel deactivates with depolarization. Similarly, if the input were to be negative that leads to a hyperpolarization. This hyperpolarization activates HCN channels, resulting in an inward current that depolarizes the neuron. Consequently, the current through HCN channels always acts to counterbalance the impact of the stimulus current.^40^ Hence, HCN channels act as a *negative feedback loop* (Figure 2A) that suppresses the voltage response irrespective of which direction the input current flows in. This counterbalancing current that flows through HCN channels implies that they suppress the steady-state voltage response, which directly translates to a reduction in input resistance and firing rate.^36–38,41,46,47^ In a spike train, this slow counterbalancing current mediated by HCN channels also allows them to mediate spike frequency adaptation. Specifically, within a spike train, cumulative deactivation of HCN channels (which are slow) by successive action potentials continually decreases the inward current, thereby increasing the interspike intervals between later spikes.

Therefore, from the perspective of HCN channel dynamics mediating a negative feedback loop, it is abundantly clear how they would be able to reduce excitability. In other words, although HCN channels mediate an inward current, this inward current is shut down by depolarization and is enhanced by hyperpolarization, mediating a biophysical mechanism that mediates a negative feedback loop that suppresses responses. Thus, ion-channel impact on excitability should not be viewed from whether a channel mediates an inward or an outward current, but on how the channel current *dynamically* varies in response to inputs in specific directions. An ion channel that shuts down an inward current in response to depolarization is a restorative conductance that reduces excitability, and not a regenerative conductance that enhances excitability.

We note that this ability to *dynamically* suppress the voltage response, and thereby reduce neuronal excitability, is simply a reflection of the unique gating properties of the HCN channels and *did not involve* any interaction with other ion channels. Thus, there are not two, but three mechanisms at play that define how HCN channels regulate excitability. The *resting* current depolarizes the RMP, the *resting* conductance reduces input resistance, and the *dynamical current response* acts as a negative feedback loop that suppresses the output (thus reducing input resistance). We will later assess the impact of other ion channels and synaptic activity on these three components when we assess interactions with other ion channels.

## 5 Dynamic Viewpoint: Sag, Rebound, and Temporal Summation

The uniqueness of HCN channels is not limited to their ability to mediate a hyperpolarization-activated inward current (Figure 1A) but also extends to the current being a slow current (Figure 1B). The slow kinetics of these currents confer upon neurons (that express these channels) important additional capabilities.^40,41,47^ First, if the channel (de)activation time constant is larger than the membrane time constant (which is typically on the order of 20–40 ms in most hippocampal and cortical neurons), HCN-induced suppression of the voltage response continues beyond the membrane reaching steady-state (Figure 2D,E). This induces a characteristic *sag potential* (Figure 2D,E) with the activation of HCN channels that deviates from the exponential-like charging that is observed in the absence of HCN channels (Figure 2B,C).^40^ Second, owing to these kinetic differences between the membrane time constant and the HCN-channel deactivation, there is also a *rebound* in the opposite direction when the current is turned off (Figure 2D,E). Sag and rebound manifest for both positive and negative stimulus directions (Figure 2D,E) and could be asymmetric depending on the direction of current (e.g., Sag and rebound in Figure 2D vs. Figure 2E), the exact membrane voltage of the neuron when the stimulus arrives, and its relationship to the activation curve (Figure 1A). The rebound in response to negative stimuli (Figure 2D), typically referred to as post inhibitory rebound, can elicit spiking if the rebound is large enough to cross action potential threshold.

Although sag and rebound are characteristic features for the expression of HCN channels, it is important to note that the absence of sag or rebound does not necessarily translate to the absence of HCN channels. As elucidated above, sag and rebound emerge as a consequence of the intricate differences between the kinetics of the ion channel and the membrane time constant. There can be scenarios where HCN channels do express, but do not result in sag or rebound because of the *relatively* faster kinetics of the channels compared to membrane time constant. In addition, interactions with other ion channels that co-express with HCN channels can also modulate the ability of neurons to manifest sag or rebound.

Although the restorative nature of HCN-channel gating properties imply that they should reduce synaptic potentials (irrespective of whether they are excitatory or inhibitory), the slow kinetics imply that they are not effective in altering the amplitude of single post-synaptic potentials (Figure 2F). Specifically, as the rise time of synaptic potentials are typically on the order of milliseconds, the slow kinetics of HCN channels translate to little time for activation/deactivation of HCN channels towards suppressing the amplitude (Figure 2F). However, as the decay time constant of synaptic potentials range from tens to hundreds of milliseconds (depending on the receptor kinetics), the suppressive capabilities of HCN channels translate to a reduction in the decay time constant of synaptic potentials, coupled with a rebound (Figure 2F). When multiple synaptic inputs arrive on the postsynaptic neuron within specific frequency ranges, the reduction in decay time constant of synaptic potentials directly translates to suppression of temporal summation with the expression of HCN channels (Figure 2F).^37,55–57^ The slow kinetics and the unique gating properties also allow HCN channels to regulate neuronal responses (such as plateau potentials and resultant dendritic spikes in neurons) elicited by coincident interactions or astrocytic activation.^58–60^

## 6 Dynamic Viewpoint: Impedance, Spectral Selectivity, Inductance, and Phase Lead

Foundational lectures in cellular neurophysiology typically start with basic elements of electrical circuit: resistors (*R*), capacitors (*C*), and inductors (*L*). In the process of introducing neural responses, lectures then delve into membrane charging curves and how neural responses could be modeled using combinations of resistors and capacitors. Students are told that the lipid bilayer in conjunction with ionic solutions on either side of the membrane together act as a capacitor, and that transmembrane proteins that are permeable to ions act as inverse resistors. An invariable question that arises during these initial classes is about whether there are inductors in neurons. As the idea of RC circuits as low pass filters is presented, a typical follow up question pertains to whether the low-pass filtering can be converted to band-pass filtering by introduction of inductive components.

Electrical equivalent circuits have been used widely in the representation and analysis of the neuronal membrane and conductances associated with it.^61–65^ Electrical impedance, defined as the ratio of the voltage response to injected current, is a standard measurement used to characterize frequency-dependent neuronal responses. Impedance, by definition, is a complex number with resistance forming its real part and reactance its imaginary part. While resistance is a positive quantity, reactance can be either positive or negative, depending on the presence of inductive or capacitive elements, respectively. Further, a reactance of zero implies that the current and voltage are in phase, while a positive or a negative reactance indicates the voltage response leading (positive phase) or lagging (negative phase) the input current, respectively. Thus, neuronal impedance forms a highly powerful and versatile measurement, as its magnitude provides a measure of frequency-dependent excitability of neurons, and its phase indicates the temporal relationship between the input current and output voltage of the neuron.^41^

The recognition of the cell membrane behaving as a resistance-capacitance (RC) circuit, and thus the presence of negative reactance as a component of its impedance occurred much before (see (Cole, 1932)^65^ and references therein) the first measurements of positive (inductive) impedance were made. The first measurement of an inductive reactance was made by Cole and Baker^62^ in the squid giant axon. They had argued against the membrane being modeled as just an equivalent RC circuit and had suggested the addition of an inductive element to the equivalent circuit.^61,62^ Cole had also suggested that such inductive elements could be realized using time-varying conductances.^66^ Later, after Hodgkin and Huxley presented their parallel conductance model,^63^ it was initially argued and later demonstrated that voltage-dependent, time-variant conductances can exhibit inherent reactive properties, which may be capacitive or inductive.^64,67–70^ It was also shown that excitatory conductance changes give rise to a capacitive reactance whereas conductance changes that aid in membrane recovery give rise to an inductive reactance.^67,69^ Such capacitive and inductive reactances emerging due to the presence of time-varying, voltage-dependent conductances have been named variously as *anomalous*^66^ or *phenomenological* reactances.^68,70^

**Figure 3:**
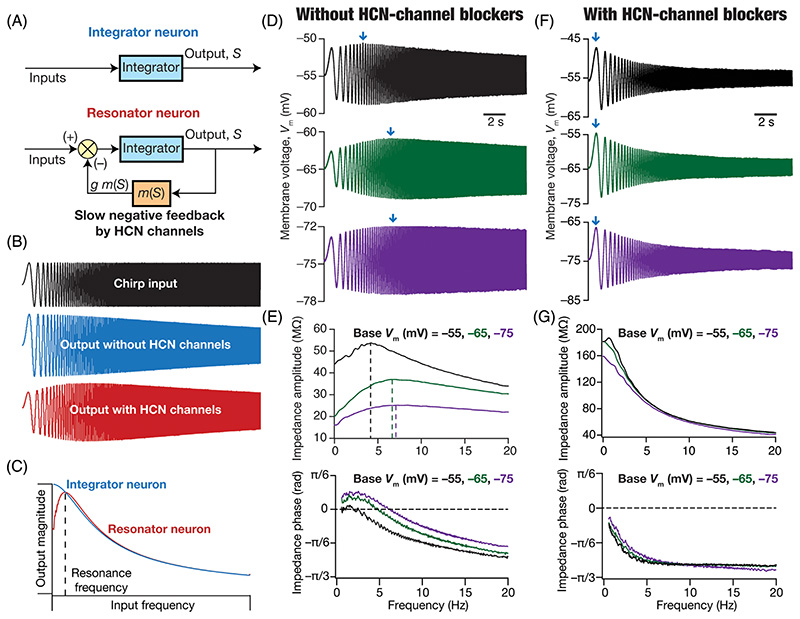
Impedance amplitude and phase profiles of neurons in the presence and absence of HCN channels. (A) *Top*, Integrator neuron. *Bottom*, Resonator neuron comprised of slow negative feedback from HCN channels. (B) Responses of neurons without (middle) and with (bottom) HCN channels to a chirp stimulus (top). (C) Response magnitude of an integrator neuron (blue) and resonator neuron (red) as functions of input frequency, derived from their respective responses to the chirp stimulus. It may be noted that the impact of HCN channels is confined to suppressing the output associated with low-frequency inputs, an observation that follows electrophysiological recordings in the presence vs. absence of HCN channels.^40,41,47^ (D) Voltage responses of a neuronal dendrite to a chirp current stimulus at different voltage values (−55, −65, and −75 mV). (E) Impedance amplitude (top) and phase (bottom) profiles derived from traces shown in panel A. The impedance amplitude profile shows resonance for all scenarios with resonance frequency increasing with hyperpolarization. The impedance phase profile shows positive phase regimes in lower frequencies, with the positive area increasing with hyperpolarization. (F) Voltage responses of a neuronal dendrite to a chirp current stimulus at different voltage values (−55, −65, and −75 mV) in the presence of a HCN channel blocker. (G) Impedance amplitude (top) and phase (bottom) profiles derived from traces shown in panel C. The impedance amplitude profile shows no resonance across all voltages. The impedance phase profile shows no positive phase regimes across all voltages. Traces in (D–G) are plotted from electrophysiological data in Narayanan and Johnston.^41,47^ Parts of panels A–C in this figure have been modified from Mittal and Narayanan.^89^

Although the use of stimuli of various frequencies in assessing neuronal impedance has been prevalent for a long time,^65,71,72^ starting in 1984,^73^ Puil and colleagues performed multiple studies on membrane resonance^74–76^ using the chirp stimulus (Figure 3B). Using chirp-current injections and various other techniques, they and others have reported electrical resonance in multiple cell types,^40,47,74–81^ and have developed analytical frameworks to analyze impedance and describe the implications of these anomalous reactances (especially inductances in conjunction with passive membrane resistance and capacitance) to filtering, resonance and oscillations.^40,68,70,75,79,82–84^ Several conductances, including those mediated by the *M*-type potassium channel, the *h* channel, *T*-type calcium channels, and some slowly activating potassium channels, have been reported to independently mediate resonance behavior in different neuronal subtypes.^40,78,80^

All resonating conductances could be considered to be equivalent to *anomalous* inductances, given that these conductances, in conjunction with the passive membrane, exhibit resonance owing to their specialized voltage- and time-dependent properties.^40^ The HCN channel is a resonating conductance, as it can elicit resonance from a passive RC circuit.^47,82^ Recognizing this, neurons that contain the *h* conductance have been modeled as equivalent RLC circuits.^40,41,85–88^

Intuitively, the emergence of resonance in the presence of HCN channels and other resonating conductances can be understood in the context of the slow negative feedback loop introduced earlier.^41,89^ The RC circuit mediates a low pass filter (Figure 3A-C). The addition of an ion channel that mediates a slow negative feedback loop to this base circuit allows targeted suppression of low-frequency inputs (Figure 3C). As the time constant associated with the feedback loop is high, there is not enough time for the channel to activate or deactivate in the presence of high-frequency inputs. On the other hand, when the inputs are in the low-frequency range, there is enough time for the channel to activate and deactivate, thereby suppressing the output voltage (negative feedback). The amount of suppression therefore is frequency-dependent, with the suppression being higher at lower frequencies. This targeted suppression of low-frequency inputs results in the emergence of resonance in these neural structures (Figure 3B,C).

The presence of HCN channels introduces membrane potential resonance in neurons, with resonance increasing with hyperpolarization of membrane voltage, making neurons behave as voltage-dependent band-pass filters (Figure 3D-G). As certain neuronal subtypes such as the hippocampal pyramidal neurons show increased expression of HCN channels in the dendrites, there is an increase in resonance frequency of dendritic compartments compared to their somatic counterparts.^47^ Expectedly, the positive phase component in the impedance phase profile also increases with membrane hyperpolarization (Figure 3E) as well as in dendritic compartments in a manner that is dependent on the current through HCN channels (Figure 3G).^41^ The presence of HCN channels in neuronal dendrites modulate local field potentials (LFP) by regulating *return currents* mediated by these ion channels.^90–93^ Importantly, the inductive phase lead in lower frequencies introduced by HCN channels contributes to spike phase differences in individual neurons with reference to the LFP.^90^

## 7 Dynamic Viewpoint: Classes of Excitability and Spike-Triggered Average

Decades ago, Hodgkin had delineated excitability into three distinct classes based on the characteristics of the *f−I* curve that defined the frequency of action potentials (*f*) elicited as a function of injected pulse current amplitude (*I*).^94^ The class of excitability was defined by how the *f−I* curve looked like beyond the rheobase current (defined as minimal current required to elicit action potential firing in a neuron). If the neuron can elicit action potentials at arbitrary small values of firing frequency, the neuron was referred to as belonging to Class 1 excitability. A neuron that elicited a single action potential for a large range of current values beyond rheobase current, and then jump to eliciting a greater number of action potentials at a high frequency belonged to Class 2 excitability. In other words, neurons belonging to Class 2 excitability cannot elicit action potentials at arbitrarily small frequency values owing to jump between single action potential regime to multiple action potential regime (at a high frequency). Neurons in the Class 3 excitability regime elicit a single action potential irrespective of how high a current is injected into the neuron.^94,95^

Although defined from the perspective of the shape of *f−I* curve, this delineation of excitability classes proved extremely insightful from several perspectives, including insights about bifurcation structures, spike initiation dynamics, neural dynamics, and neural coding. The need to account for the class of excitability of a neuron also arose in defining what neurons are capable of, with coincidence detection and temporal coding at the very top of the list.^95–110^ Specifically, neurons endowed with Class 1 excitability characteristics act as *integrators*, whereas neurons with Class 2/3 excitability act as *coincidence detectors*. Much of the controversy associated with whether neurons are equipped to perform coincidence detection arise from considering neurons as Class 1 integrators without accounting for the two other classes of neurons that are capable of performing coincidence detection with high precision.^98–100,111–114^

The classes of excitability have been defined in terms of the competition between slow peri-threshold restorative vs. regenerative currents.^95^ Although the distinction has been thought of as in terms of outward vs. inward currents, we argue that the restorative vs. regenerative distinction is more apt as a channel that mediates a net inward current could act as a restorative conductance (e.g., HCN channels; Figure 2A) Specifically, a neuron endowed with a net slow regenerative conductance manifests Class 1 excitability whereas neurons where the restorative slow conductance wins the competition displays Class 2/3 excitability.^95,98–100,114^ As HCN channels mediate an important slow restorative conductance that is widely expressed in neurons, their expression profile can alter the class of excitability of neurons. Neurons with high density of HCN channel expression can act as coincidence detectors because the presence of HCN channels allows the slow restorative current to dominate peri-threshold dynamics.^98–100,114^

The three different classes of excitability can be considered as a continuum, with expression profiles of resonating conductances such as HCN channels allowing neurons to traverse this continuum. This continuum of classes of excitability also represents the integrator– coincidence detector continuum, whereby a neuron can traverse back-and-forth between being an integrator to being an ideal coincidence detector simply by adjusting the density of specific sets of ion channels.^98–100,114,115^ HCN channels are one among a class of such ion channels whose density can define the operating regime of the neuron as an integrator or a coincidence detector.

The spike-triggered average (STA) and measures such as the STA characteristic frequency and coincidence detection window can be used as graded measures of switches across the different classes of excitability rather than viewing them as three distinct discrete classes.^98–100,108,114,115^ To elaborate, STA defines the average stimulus that triggers a spike in a neuron. The characteristics of STA are strongly dependent on the properties of the ion channels expressed by the neuron.^95,98–100,104–108,114–117^ Class 1 neurons show uniphasic STA defining time-dependent integration of incoming stimuli. In contrast, Class 2/3 neurons manifest biphasic STA with a spike-proximal positive lobe and a negative lobe that together govern coincidence detection, with the negative lobe also indicative of post-inhibitory rebound capabilities of Class 2/3 neurons.^95,98–100,114^ Among other routes to switch along the integrator-coincidence detector continuum, increase in HCN-channel density can switch the STA shape across classes. The measures from the STA therefore span a continuum of values indicative of such switches across classes with changes in HCN-channel density.^98–100^

## 8 Spatial Viewpoint: Implications for Inhomogeneous Distribution of HCN Channels

There are lines of evidence from different neuronal subtypes for the expression of HCN channels across all compartments. Apart from their expression in the soma and dendrites of several neurons,^36–38,56,112,118–123^ they also express in axonal initial segments^124,125^ and in synaptic terminals.^126^ An important attribute of HCN channels is their inhomogeneous distribution within several neurons, especially cortical and hippocampal pyramidal neurons where HCN-channel density is higher in distal dendritic compartments. There have been several functional roles attributed to such inhomogeneous distribution of HCN channels in pyramidal neurons.

As a direct consequence of the resting inward current mediated by HCN channels, the resting membrane potential of distal dendritic locations is depolarized compared to the somata and the proximal dendritic locations.^127^ As a consequence of the gradient in HCN-channel density, the resting conductance as well as the ability to suppress the steady-state responses (as a consequence of a stronger negative feedback loop) are higher in distal dendritic compartments. This results in a reduction in input resistance in distal compartments compared to their proximal counterparts in hippocampal and cortical pyramidal neurons.^37,47,128^ Thus, higher expression of HCN channels in distal compartments results in a more depolarized RMP and reduced input resistance values in these compartments.

An important functional role for a distance-dependent increase in HCN channel density is the ability of such inhomogeneous distribution to normalize temporal summation across the somato-dendritic axis.^55,56,129^ Specifically, passive cable filtering increases the rise time and the decay time constant of synaptic potentials.^130–132^ Thus, if the input characteristics of synaptic potentials are identical across locations, distal synaptic potentials incur larger attenuation and filtering as they traverse a larger distance to the soma compared to their proximal counterparts. Therefore, somatically measured temporal summation for proximal synaptic inputs will be lesser compared to that of the distal synapses, because the somatically measured decay time constant of distal synaptic potentials will be higher. HCN channels, however, reduce temporal summation as a function of their ability to act as slow negative feedback loops. Thus, adjusting the density of HCN channels in distal locations to be higher compared to proximal dendritic locations would yield a larger reduction in local temporal summation at distal locations. The enhancement in temporal summation induced by cable filtering can thus be nullified by a location-dependent suppression of temporal summation with HCN channels expressing at higher densities at distal locations. There are experimental lines of evidence to suggest that the manifestation of a positive proximo-distal gradient in HCN channels normalizes temporal summation across the somato-dendritic arbor.^55,56,129^

From the perspective of impedance measurements, the inhomogeneous distribution of HCN channels in pyramidal neurons mediate functional maps in maximal impedance amplitude, resonance frequency, resonance strength, and inductive phase component as functions of somato-dendritic distance. Whereas resonance frequency, resonance strength, and inductive phase increase as functions of dendritic distance from the soma, there is a reduction in the maximal impedance amplitude along the same axis.^41,47,128,133,134^ The gradient in impedance phase introduced by the inhomogeneous distribution of HCN channels also produces temporal synchrony (at the somata) of rhythmic inputs impinging on different dendritic locations.^135^ Akin to the normalization of temporal summation, the differential positive phase introduced by inhomogeneous distribution of HCN channels counteracts the negative phase introduced by distance-dependent cable filtering to elicit somatic temporal synchrony.^135^ The impact of inhomogeneous distribution of HCN channels on impedance measurements and other properties also depend on morphological and electrical characteristics of the somato-dendritic structure.^114,136,137^

Whereas impedance measurements relate to subthreshold excitability, the inhomogeneous distribution of HCN channels also have a pronounced impact on the STA and coincidence detection windows.^98–100,114^ Specifically, the STA of individual neurons transitioned from Class I excitability at proximal dendritic locations to Class II/III for distal dendritic locations, demonstrating that STA should be considered as a compartment-specific measurement rather than a neuron-specific measurement. Owing to this transition, the STA characteristic frequency and the STA selectivity strength increased with increasing distance. Importantly, owing to the inhomogeneous distributions of ionic conductances, coincidence detection window reduced from the slow gamma range (25–60 Hz) in proximal locations to the fast gamma range (60–150 Hz) in the distal locations,^99,100,114^ matching with the specific gamma range inputs they have been shown to be associated with.^138–141^

## 9 Interactions Viewpoint: Dependence on Co-Expressing Channels

The impressive cellular physiological abilities of HCN channels are further amplified when they naturally interact with the several other ion channels that express in neurons and their dendrites. The most prominent of all the outstanding functions that are possible because of HCN channels interacting with other channels is their pacemaking capability. HCN channels are also called as pacemaking channels and require either noise or interaction with other ion channels for manifesting sustained oscillations. HCN channels can introduce damped oscillations in neurons based on their expression profile and their kinetics. The voltage sag that is observed in the presence of HCN channels (Figure 2D,E) is just the first cycle of a damped oscillation.^40^

As a biophysical mechanism for mediating slow negative feedback loop (Figure 2A), it is theoretically clear that HCN channels should be able to maintain oscillations with either noise or with an additional fast mechanism that can sustain damped oscillations introduced by HCN channels.^142^ It has been shown using electrophysiological and computational techniques that HCN channels can indeed act as pace-making channels when they interact with either noise or with other fast conductances that amplify the damped oscillations.^1–4,40,108,143–153^ HCN channels are critically involved in pacemaking physiology in several neuronal subtypes across the brain, especially in mediating low-frequency oscillations typically in the theta-frequency (3–10 Hz) range.^2,3,5,153–155^

Each of the several functional properties mediated by HCN channels can be modulated by the presence of and interactions with other ion channels and receptors. Under resting conditions, the conductance-current conundrum presented by the expression of HCN channels is strongly tempered by the expression of other ion channels that are also active under resting conditions. Specifically, much of the debate about the conductance-current conundrum stems from considering HCN channels in isolation with the passive properties of the neuron (apart from not accounting for the dynamic nature of HCN channel gating kinetics resulting in a negative feedback loop). However, neuronal physiology emerges because of strong interactions between the several sub- and supra-threshold ion channels that express across the spatio-temporal extent of neuronal membrane. Thus, we argue that the very question of whether a given ion channel enhances or reduces excitability is ill posed without the context of what other channels are present and how they interact with the considered ion channel.^42,45,54^

**Figure 4:**
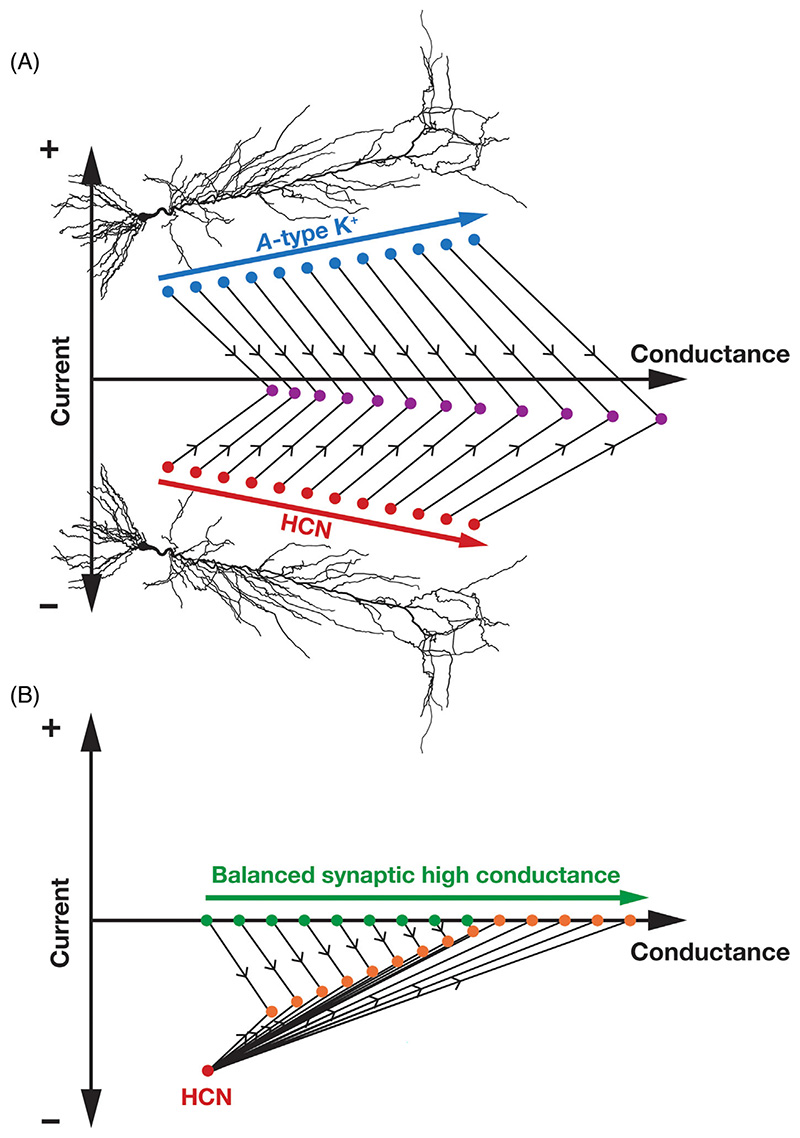
Pictorial representation of the impact of ion channel interactions inspired by Feynman diagrams for interactions among subatomic particles. (A) HCN channels (red circles) mediate inward (negative) currents and contribute to an increased conductance. *A*-type K^+^ channels (blue circles) mediate outward (positive) currents and contribute to an increased conductance. When they are co-expressed, they interact with each other (black lines), ensuring that the conductance-current balance is titled in favor of conductance (purple circles depict larger values on the conductance axis, but have smaller values on the current axis). Both HCN and *A*-type K^+^ channels increase along the somato-apical trunk of hippocampal CA1 pyramidal neurons, leading to an increase in both conductance and currents along this axis. Their interactions (black lines) suppress the impact of current and enhance the impact of conductance thereby leading to an overall restorative influence along the somatodendritic axis. Interactions between HCN and *A*-type K^+^ channels have been electrophysiologically demonstrated using pharmacological agents.^158^ (B) The presence of an HCN-channel cluster (red circle) results in an inward (negative) current and contributes to an increased conductance. However, when balanced synaptic high-conductance states are coexistent (green circles), they progressively suppress the impact of HCN channels on both conductance and current (orange circles) based measurements. The different orange circles refer to progressively larger number of synapses, and the balanced nature of synaptic inputs ensures that these circles lie on the conductance axis with no impact on average resting potential. Whereas *A*-type K^+^ channels tilt the HCN conductance-current balance heavily in favor of conductance (A), the impact of HCN channels on conductance- and current-based measurements are severely weakened under synaptically driven high-conductance states (B). These diagrams summarize findings in Mishra and Narayanan.^54^

For instance, in the presence of co-expressing *A*-type K^+^ channels, the net effect of HCN channels is a *suppression* of the current-based impact with conductance-based impact playing a dominant role (Figure 4A). Specifically, both HCN and *A*-type K^+^ channels show an increasing gradient in their expression profile along the somato-apical axis of hippocampal pyramidal neurons.^37,156^ The interaction between these two subthreshold ion channels yields a scenario where the current-based impact is suppressed by the correlated expression profiles of one inward (HCN) and another outward (*A*-type K^+^) current (Figure 4A). On the other hand, as both channels yield an enhanced conductance-based impact owing to their ability to reduce input resistance,^36,37,47,157,158^ there is a dominance of conductance-based impact (Figure 4A).^54^ The *A*-type K^+^ channels are fast depolarization-activated inactivating channels whose kinetics and gating profile are very different from the slow hyperpolarization-activated HCN channels. However, the window component associated with *A*-type K^+^ channels in hippocampal pyramidal neurons spanning the subthreshold voltage regime^156,157,159–161^ allow for an overlapping voltage range where these two conductances could interact.^54^ The ability of *A*-type K^+^ channels to alter input resistance,^157,158^ a steady-state measurement that typically does not depend on inactivating channels, is testament to the impact of the critical role played by the window component on neuronal physiology.

Under *in vivo* conditions where the background synaptic activity is high (Figure 4B), the presence of balanced high-conductance states (where the average net current flowing is zero, while the synaptic conductance being high contributes to reduced input resistance) play a critical role in regulating the conductance-current balance.^54^ Specifically, in the presence of high-conductance states, the impact of HCN current is suppressed because of the gain of the neuron is reduced by the high-conductance state. In addition, when the background activity is extremely high, the parallel conductance mediated by the background activity dominates over the HCN (or other channel) conductances which have a relatively lower conductance value compared to the net synaptic conductance (Figure 4B). Thus, it is critical that the debate on the conductance-current balance explicitly accounts for interactions of HCN channels with other ion channels and receptors and their subthreshold expression and activation profiles.^42,45,54^

Similarly, although HCN channels mediate sag, post-inhibitory rebound, membrane potential resonance, coincidence detection, and oscillations in neurons, these physiological outcomes can be critically regulated by other co-expressing ion channels.^98–100,108,114,115,147,158,162–168^ As an example, the absence of sag or resonance thus should not be construed as evidence for the absence of HCN channels (or other resonating conductances) because resonance/sag could be suppressed by interactions with other co-expressing channels. To elaborate, the question of the relationship between a given ion channel and any of these physiological properties assumes a unique one-to-one mapping between ion channels and physiological outcomes. However, in the context of diversified ion-channel expression profiles and the *global structure* of their cross-interactions, neuronal functions emerge from a many-to-many relationship between ion channels and physiology.^162,163^ Thus, the evaluation of the role of HCN channels (or any other channel) on neuronal physiological characteristics must account for the global structure of ion-channel expression in that specific neuron and for neuron-to-neuron variability in terms of how each ion channel contributes to different measurements.^54,108,147,162,165,166,168–171^

HCN channels expressed in the presynaptic compartments have been shown to interact with calcium channels to regulate synaptic release properties. Specifically, the depolarization caused by HCN channels to the resting potential of the presynaptic terminal acts to govern inactivation of calcium channels in the presynaptic terminal.^12,126^ It is important that this impact of HCN channels on calcium influx and synaptic release is also critically reliant on the global structure and properties of the specific channels expressed in the presynaptic compartment.^172^ A similar analysis on the important role of interactions of HCN channels with spike-generating and other conductances holds for their specific roles in axonal initial segment as well.^124,125^ Specifically, the different ion channels and receptors expressed in these compartments and their interactions with HCN channels should be accounted for in assessing their impact on conductance-current balance and other physiological outcomes.

How do we characterize and report physiological properties in a scenario where changes to a channel alters several properties, especially resting membrane potential, through interactions with other ion channels? While there are no perfect answers to this, we have argued for the importance of measuring and reporting physiological quantities at multiple voltages.^41,47,54,100,108,147,158,165,169,173^ To elaborate, if one is interested in measuring resonance in the presence vs. the absence of HCN channels (Figure 3), the situation becomes tricky because blockade of HCN channels also depolarizes the membrane. One way to assess the impact of HCN-channel blockade is to perform resonance measurements at the respective membrane voltages before and after blockade. However, as the depolarization induced by HCN-channel blockade can be as large as 10 mV, this implies a change in driving force not just for HCN channels but for all channels across the different compartments. In addition, as conductance-current balance is modulated by other ion channels and by background synaptic activity (Figure 4), the depolarization observed under *in vitro* conditions is not representative of what could be happening *in vivo*. In such a scenario, measuring resonance properties before and after at fixed voltages offers an ideal way to compare the impact of HCN channel blockade on resonance properties (Figure 3). Thus, in reporting physiological characteristics of neurons where membrane potential changes are observed, we suggest reporting of measurements at multiple voltages and not just one single voltage in addition to reporting the membrane potential changes themselves.

## 10 Plasticity Viewpoint: Homeostasis, Metaplasticity, and Pathophysiology

**Figure 5:**
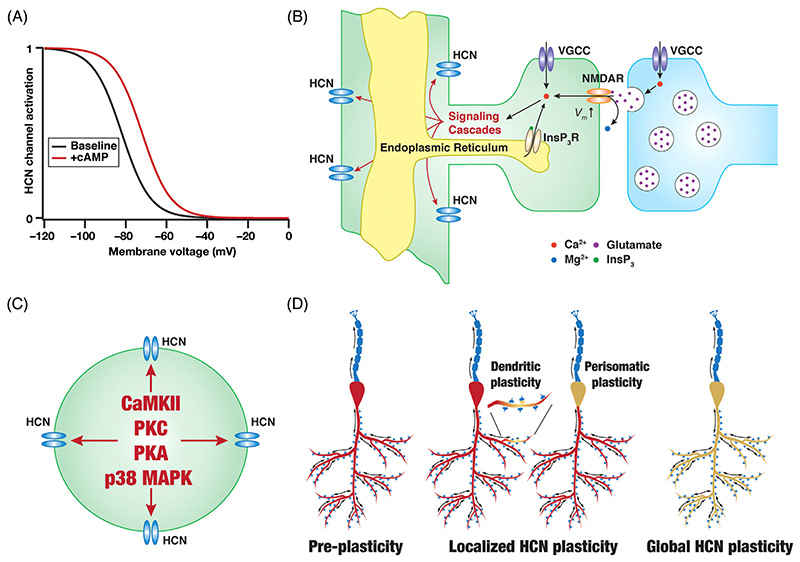
Modulation and plasticity of HCN channels. (A) Illustration of the shift in the activation profile of HCN channels by cyclic AMP (+cAMP) with reference to baseline condition without cAMP binding (baseline). A rightward shift in the activation profile may be observed in the presence of cAMP.^1,4,151,192^ The amount of cAMP-induced shift in activation profile is dependent on the specific subunits that the HCN-channel is constructed with. (B) Illustration of various calcium sources that have been implicated in HCN-channel plasticity. Calcium entry into the cytosol through NMDAR,^46,47^ voltage-gated calcium channels (VGCC),^185,187^ or inositol trisphosphate receptors (InsP_3_R)^173,187^ can activate downstream signaling cascades towards inducing HCN-channel plasticity. (C) Illustration showing some of the prominent signaling mechanisms that have been implicated in HCN plasticity: CaMKII,^46,190^ PKA,^173,187^ PKC,^188^ p38 MAPK.^189,200^ (D) Plasticity in HCN channels can be either localized to dendrites/soma^173,184^ or be spatially widespread.^41,47,188^ Panel D was modified from Figure 2 of Mishra and Narayanan.^178^ HCN-channel plasticity with different spatial localization profiles and the role of these different calcium sources and enzymes in mediating HCN-channel plasticity have been experimentally demonstrated.

Plasticity in the brain is ubiquitous^123,133,174–182^ and HCN channels are no exception in their ability to undergo bidirectional plasticity. Changes in HCN-channel properties as a function of cyclic nucleotide binding is central to HCN-channel function and is a target for several neuromodulatory influences on HCN channels (Figure 5A). As mentioned earlier, the dependence of HCN channels on cAMP is differential across the different HCN-channel subunits.^14,16,19–23^ Thus, cAMP-dependence in native HCN channels would critically rely on the relative expression of the different subunits in the neuron under consideration.^20,24,25^ In addition, calcium influx through NMDA receptors, voltage-gated ion channels, and calcium channels on the endoplasmic reticular membrane have been shown to be involved in HCN-channel plasticity (Figure 5B). In terms of downstream signaling cascades, several kinases including CaMKII, PKC, PKA, and p38 MAPK have been implicated in regulating HCN-channel function (Figure 5C). Mechanistically, HCN-channel plasticity could involve changes in the gating properties of the channels and/or changes to the surface expression and binding profiles of channel subunits. Importantly, HCN-channel plasticity can be spatially localized to specific regions of dendrites/perisomatic regions or can be spatially widespread across the dendritic tree (Figure 5D). Together, the modulatory and plasticity repertoire associated with HCN channels is extensive and has been implicated in several physiological and pathophysiological roles across different brain regions.^1,3,5–12,24,41,43,46,47,150,151,173,183–195^

As a direct consequence of the ability of HCN channels to mediate a negative feedback loop (Figure 2A), it is unsurprising that plasticity resulting in an increase in the postsynaptic current mediated by these channels has been interpreted as a homeostatic and neuroprotective mechanism.^41,46,47,89,133,164,165,173,188,196–199^ Along similar lines, loss of HCN channels has been shown to result in hyperexcit-ability and a loss of homeostatic maintenance.^9,11,12,26,60,200–204^

An interesting scenario for HCN-channel plasticity associated with bidirectional synaptic plasticity has led to a postulate for HCN channels to act as a candidate mechanism for the sliding modification threshold in hippocampal pyramidal neurons.^133,197,205^ Specifically, the BCM-like plasticity rule that has been used as a theoretical framework for understanding hippocampal synaptic plasticity envisages an activity-dependent sliding modification threshold that determines the transition from potentiation to depression.^206–208^ As this sliding modification threshold follows postsynaptic activity, the threshold value increases with long-term potentiation (LTP) and reduces with long-term depression (LTD). Importantly, this change in threshold for plasticity must be common for all synapses in the neuron. Although several mechanisms have been proposed for the regulation of this sliding modification threshold,^208–211^ HCN channels are an attractive target to regulate the sliding modification threshold. First, LTP in hippocampal neurons is accompanied by an increase in HCN channel activity^46^ and LTD is accompanied by a reduction.^188^ Second, bidirectional plasticity in HCN channels associated with LTP and LTD are spatially widespread and are not limited to only the channels closer to the synapses undergoing plasticity.^47,188^ Third, and importantly, as a direct consequence of the ability of HCN channels to suppress voltage responses and reduce temporal summation (Figure 2), increase in the current through HCN channels causes an increase in the sliding modification threshold.^205^ Together, HCN plasticity implies that LTP is associated with an increase in the sliding modification threshold whereas LTD is accompanied by a reduction, the precise scenario envisaged by the BCM plasticity rule. Thus, HCN plasticity, apart from acting as a homeostatic mechanism could also be a mechanism underlying the sliding modification threshold.^133,197,205^ A calcium-dependent plasticity rule for HCN channels has been proposed, and in conjunction with calcium-dependent synaptic plasticity can provide stability and homeostasis in model neurons.^197^

Although plasticity in HCN channels has been shown to have specific implications to neuronal physiology and synaptic plasticity profiles, it is extremely critical that plasticity in HCN channels is not treated in isolation disregarding other changes that co-occur in different cells and their circuits. Neural activity, depending on temporal patterns and levels of activity, recruits several signaling components and can concomitantly alter several molecular components with very different spatial and temporal profiles. For instance, theta-burst pairing (TBP) in hippocampal CA1 pyramidal neurons concomitantly increases synaptic strength,^212^ locally reduces current through *A*-type^159,213^ and calcium-dependent potassium^214^ channels, and globally enhances HCN-channel current^46,47^ by recruiting different downstream signaling cascades.^178^ Thus, measuring only synaptic strength would have given a false impression that TBP is a synaptic plasticity protocol, but measurement of all components show that these components change in *specific* directions in a manner that is strongly coupled to each other.

The ubiquitous nature of plasticity does not imply that plasticity is arbitrary, either in terms of the specific components undergoing plasticity under a given condition or in the sign of such plasticity. The strong structure imposed by signaling cascades on *concomitant* plasticity thus makes combined plasticity to fall within a structured *plasticity manifold*.^178,215^ There is a critical need to therefore not just account for the global structure of ion channels in neurons, but also to track plasticity across all components across different time scales to specifically understand the impact of plasticity in HCN channels in the context of plasticity in all the other components. Such analyses should also account for plasticity heterogeneity that is observed across all plasticity induction protocols, and the ability of disparate components to elicit the same plasticity profile.^178,205,209,216,217^ Tracking plasticity in any one component (such as synaptic weight or HCN channels) and interpreting outcomes based on changes in only that single component would therefore result in erroneous conclusions that do not respect the holistic impact of all changes that are concomitant to plasticity in that single component. Experimental and theoretical analyses need to account for plasticity across all components before adjudging the impact of any form of plasticity on different aspects of neural and network function.

HCN-channel plasticity has been observed in several cardiac and neurological disorders as well, contributing to several instances of HCN channels acting as effective drug targets.^8,9,11,198,203,218^ Several anesthetics and anticonvulsants have been shown to modulate HCN channels.^11,57,198,203,219–225^ HCN channel plasticity has been observed in several neurological disorders including chronic stress and depression,^12,51,203,226–229^ epilepsy,^6,8,9,11,52,57,60,189,198,200–202,204,230–235^ Alzheimer’s disease,^9,236,237^ and Fragile X syndrome.^199,238–241^ Even in the scenario of neurological disorders, it is essential to account for the heterogeneities across individual animals in terms of how they respond to treatments, the global structure of different ion channels, and plasticity spanning all components across all cellular compartments in effectively adjudging the impact of HCN channel changes on disease etiology.

## 11 Viewpoint on the Future: HCN Channels and their Interactions in Vivo

As is evident from the synthesis presented above, impressive strides have been achieved in our understanding of the cellular physiological roles of HCN channels. There is abundant clarity on the specific roles of HCN-channel physiology and plasticity (as well as their interactions with other cellular components and their plasticity) on neuronal physiology. We envisage this clarity as a definite first step towards understanding the multifarious roles of this enigmatic channel on multi-scale physiology *in vivo*. To elaborate, much of our understanding about HCN channels, their physiology and plasticity come from *in vitro* experiments and are mostly confined to critical insights into cellular aspects of neural function. However, these insights do not offer clarity about the specific roles of HCN channels under *in vivo* conditions, wherein neurons receive behavior-driven activity patterns. Activity profiles of different ion channels, with expression patterns specific to individual neurons, then participate in sculpting neuronal firing patterns, network activity, and behavioral activity. Thus, the critical question for the future is about how HCN channels and their interactions with other channels drive multi-scale activity (spanning from neurons to behavior) under *in vivo* conditions. In what follows, we present certain potential directions where HCN-channel function could be probed *in vivo*.

It is essential to address the question of how plasticity in HCN channels, coupled to interactions with plasticity in other components, drive learning and memory. Specifically, the role of ion-channel plasticity on engram cell formation is now clearly established. As a dominant channel that strongly regulates sub- and supra-threshold excitability, HCN channels are ideally placed to play critical roles in engram cell formation.^185^ Future experiments could focus on developing techniques for imaging HCN channels during learning tasks in a manner similar to how AMPARs have been imaged *in vivo*.^242–244^ Novel techniques need to be developed to directly record changes in conductance values and gating properties of HCN channels from different neuronal compartments *in vivo* during learning tasks. In addition to such *in vivo* analysis, available super resolution microscopy techniques^245^ should be employed to assess the nano-domain organization of HCN channels and their interactions with other molecules in different neuronal compartments under different conditions. Given the widespread roles of HCN channels in several pathological conditions, such analyses should be repeated with different disease models, together providing deeper insights about impairments to HCN-channel function and plasticity in different disorders. In this context, species differences in HCN-channel properties, expression profiles, and plasticity need to be carefully catalogued before conclusions are generalized across different species.^246,247^

Most analyses associated with HCN channels focus on their ability to alter neural excitability or to act as pacemakers. While pacemaking and excitability are extremely important physiological characteristics, the multifarious roles of HCN channels in altering other aspects of neuronal function warrants a deeper exploration on how they contribute to multi-scale function. Future *in vivo* exploration should extend focus to the ability of HCN channels to alter the class of excitability of individual compartments,^98–100^ to mediate coincidence detection,^99,100,112,114^ to suppress low-frequency signals and noise.^40,41,47,89^ Importantly, focus should also be on the ability of cells to *titrate* each such characteristic by altering HCN-channel properties and their interactions with other channels. Such analysis under *in vivo* conditions would provide important insights about how HCN channels contribute to cellular, network, and behavioral function under different contexts.

*Interactions* among different components and their plasticity mediate all aspects of physiology. For instance, interactions among different ion channels mediate cellular neurophysiology,^63,162,163^ interactions among different neurons provide a population code of environmental variables,^248–250^ interactions among different brain regions are linked to behavioral outcomes,^251–254^ and interactions among plasticity in different components mediate multi-scale stable continual learning.^178,181,255^ In deciphering the implications for such complex interactional space on multi-scale physiology, it is essential to assess the contributions of individual components as well as variability in such contributions. Given the central role of HCN channels in regulating several aspects of neuronal physiology, it would be critical to understand how changes in their properties alter multi-scale physiology through such interactions.

For instance, how do neural manifolds and population representations that are associated with a network change when there is plasticity in HCN-channel density or properties within the neurons that form this network? The complexities associated with addressing this question are enormous as plasticity in different neuronal subtypes could go in different directions and there could be neuron-to-neuron variability in changes to the HCN-channels. The implications for such changes to the neural manifold or population representation would depend on several factors. Some such factors are the network architecture, the pattern and intensity of afferent activity, local synaptic weight strengths, intrinsic properties of different neurons, the exact nature of plasticity in HCN channels in each neuron, and interactions among molecular components and their plasticity in different neurons. Future studies could systematically focus on delineating the impact of HCN channels and their plasticity on neural manifolds and population representations of environmental variables. Such analyses could act as a steppingstone towards understanding the multi-scale impact of HCN-channel plasticity.

## 12 Conclusions

The hyperpolarization-activated cyclic-nucleotide-gated ion channel, the mediator of the caricaturized “funny” current, possesses idiosyncratic characteristics that bestow unique physiological capabilities upon cells they express in. Unique structural characteristics of the HCN proteins allow these ion channels to be activated when the membrane potential becomes more hyperpolarized, unlike most voltage-gated ion channels that open with depolarization. Adding to this unique gating property are other characteristics, namely their non-specific permeability to monovalent cations and their slow kinetics spanning tens to thousands of milliseconds. The net inward current through HCN channel leads to depolarization of membrane potential, taking the neuron closer to its firing threshold. Contrary to this, the expression of HCN channel enhances the overall resting conductance and reduces the excitability of a neuron. This results in the conductance-current conundrum associated with the expression of HCN channels that has been central to several debates about their physiological roles.

In this synthesis exploring the cellular physiological role of HCN channels, we argued that this conundrum is simply consequent to viewing HCN channels from a static resting viewpoint expressed in isolation. Instead, we posit that the role of HCN channels in regulating neuronal physiology is better understood when HCN channels are visualized from a dynamic viewpoint, while also accounting for other co-expressing ion channels. From a dynamical viewpoint, we discussed how HCN channels in response to *time-varying* inputs (rather than under steady-state conditions) mediate a *slow negative feedback loop* that suppresses neuronal responses irrespective of which direction the input current flows in. We present the several physiological implications of this negative feedback loop to neuronal response characteristics including gain (of voltage response and action potential firing), sag, rebound, temporal summation, resonance, inductive phase lead, and coincidence detection. From an interactions standpoint, we argue that the impact of HCN channels on cellular physiology should account for other co-expressing ion channels, each of which (including HCN channels) are typically endowed with heterogeneous distributions in different neuronal subtypes. Through various illustrative examples from different brain regions, we argue why it is crucial to consider the presence of co-expressing ion channels and relative distribution while assessing the impact of HCN channels on neuronal response properties. Finally, we discuss how plasticity in HCN channels is widely prevalent and can mediate different encoding, homeostatic, and neuroprotective features in a neuron depending on the context and global structure associated with HCN-channel plasticity.

## Data Availability

Data sharing not applicable to this article as no datasets were generated or analysed during the current study.

## References

[1] DiFrancesco D (2019). A brief history of pacemaking. Front Physiol.

[2] Robinson RB, Siegelbaum SA (2003). Hyperpolarization-activated cation currents: from molecules to physiological function. Annu Rev Physiol.

[3] Pape HC (1996). Queer current and pacemaker: the hyperpolarization-activated cation current in neurons. Annu Rev Physiol.

[4] DiFrancesco D (2010). The role of the funny current in pacemaker activity. Circ Res.

[5] Combe CL, Gasparini S (2021). I(h) from synapses to networks: HCN channel functions and modulation in neurons. Prog Biophys Mol Biol.

[6] Crunelli V, David F, Morais TP, Lorincz ML (2023). HCN channels and absence seizures. Neurobiol Dis.

[7] Hennis K, Biel M, Wahl-Schott C, Fenske S (2021). Beyond pacemaking: HCN channels in sinoatrial node function. Prog Biophys Mol Biol.

[8] Rivolta I, Binda A, Masi A, DiFrancesco JC (2020). Cardiac and neuronal HCN channelopathies. Pflugers Archiv.

[9] Santoro B, Shah MM (2020). Hyperpolarization-activated cyclic nucleotidegated channels as drug targets for neurological disorders. Annu Rev Pharmacol Toxicol.

[10] Chang X, Wang J, Jiang H, Shi L, Xie J (2019). Hyperpolarization-activated cyclic nucleotide-gated channels: an emerging role in neurodegenerative diseases. Front Mol Neurosci.

[11] Brennan GP, Baram TZ, Poolos NP (2016). Hyperpolarization-activated cyclic nucleotide-gated (HCN) channels in epilepsy. Cold Spring Harb Perspect Med.

[12] Shah M (2018). Neuronal HCN channel function and plasticity. Curr Opin Physiol.

[13] Ludwig A, Zong X, Jeglitsch M, Hofmann F, Biel M (1998). A family of hyperpolarization-activated mammalian cation channels. Nature.

[14] Ludwig A, Zong X, Stieber J, Hullin R, Hofmann F, Biel M (1999). Two pacemaker channels from human heart with profoundly different activation kinetics. EMBO J.

[15] Ishii TM, Takano M, Ohmori H (2001). Determinants of activation kinetics in mammalian hyperpolarization-activated cation channels. J Physiol.

[16] Ishii TM, Takano M, Xie LH, Noma A, Ohmori H (1999). Molecular characterization of the hyperpolarization-activated cation channel in rabbit heart sinoatrial node. J Biol Chem.

[17] Santoro B, Liu DT, Yao H (1998). Identification of a gene encoding a hyperpolarization-activated pacemaker channel of brain. Cell.

[18] Santoro B, Chen S, Luthi A (2000). Molecular and functional heterogeneity of hyperpolarization-activated pacemaker channels in the mouse CNS. J Neurosci.

[19] Seifert R, Scholten A, Gauss R, Mincheva A, Lichter P, Kaupp UB (1999). Molecular characterization of a slowly gating human hyperpolarization-activated channel predominantly expressed in thalamus, heart, and testis. Proc Natl Acad Sci U S A.

[20] Altomare C, Terragni B, Brioschi C (2003). Heteromeric HCN1-HCN4 channels: a comparison with native pacemaker channels from the rabbit sinoatrial node. J Physiol.

[21] Wainger BJ, DeGennaro M, Santoro B, Siegelbaum SA, Tibbs GR (2001). Molecular mechanism of cAMP modulation of HCN pacemaker channels. Nature.

[22] Stieber J, Stockl G, Herrmann S, Hassfurth B, Hofmann F (2005). Functional expression of the human HCN3 channel. J Biol Chem.

[23] Mistrik P, Mader R, Michalakis S, Weidinger M, Pfeifer A, Biel M (2005). The murine HCN3 gene encodes a hyperpolarization-activated cation channel with slow kinetics and unique response to cyclic nucleotides. J Biol Chem.

[24] Chen S, Wang J, Siegelbaum SA (2001). Properties of hyperpolarization-activated pacemaker current defined by coassembly of HCN1 and HCN2 subunits and basal modulation by cyclic nucleotide. J Gen Physiol.

[25] Ulens C, Tytgat J (2001). Functional heteromerization of HCN1 and HCN2 pacemaker channels. J Biol Chem.

[26] Shah MM (2014). Cortical HCN channels: function, trafficking and plasticity. J Physiol.

[27] Notomi T, Shigemoto R (2004). Immunohistochemical localization of Ih channel subunits, HCN1-4, in the rat brain. J Comp Neurol.

[28] Lee CH, MacKinnon R (2017). Structures of the human HCN1 hyperpolarization-activated channel. Cell.

[29] Lee CH, MacKinnon R (2019). Voltage sensor movements during hyperpolarization in the HCN channel. Cell.

[30] Santoro B, Baram TZ (2003). The multiple personalities of h-channels. Trends Neurosci.

[31] Santoro B, Piskorowski RA, Pian P, Hu L, Liu H, Siegelbaum SA (2009). TRIP8b splice variants form a family of auxiliary subunits that regulate gating and trafficking of HCN channels in the brain. Neuron.

[32] Ulens C, Siegelbaum SA (2003). Regulation of hyperpolarization-activated HCN channels by cAMP through a gating switch in binding domain symmetry. Neuron.

[33] Goldman DE (1943). Potential, impedance, and rectification in membranes. J Gen Physiol.

[34] Hodgkin AL, Katz B (1949). The effect of sodium ions on the electrical activity of giant axon of the squid. J Physiol.

[35] Johnston D, Wu SM (1995). Foundations of Cellular Neurophysiology.

[36] Gasparini S, DiFrancesco D (1997). Action of the hyperpolarization-activated current (Ih) blocker ZD 7288 in hippocampal CA1 neurons. Pflugers Archiv.

[37] Magee JC (1998). Dendritic hyperpolarization-activated currents modify the integrative properties of hippocampal CA1 pyramidal neurons. J Neurosci.

[38] Maccaferri G, Mangoni M, Lazzari A, DiFrancesco D (1993). Properties of the hyperpolarization-activated current in rat hippocampal CA1 pyramidal cells. J Neurophysiol.

[39] He C, Chen F, Li B, Hu Z (2014). Neurophysiology of HCN channels: from cellular functions to multiple regulations. Prog Neurobiol.

[40] Hutcheon B, Yarom Y (2000). Resonance, oscillation and the intrinsic frequency preferences of neurons. Trends Neurosci.

[41] Narayanan R, Johnston D (2008). The h channel mediates location dependence and plasticity of intrinsic phase response in rat hippocampal neurons. J Neurosci.

[42] Migliore M, Migliore R (2012). Know your current I(h): interaction with a shunting current explains the puzzling effects of its pharmacological or pathological modulations. PLoS One.

[43] Rosenkranz JA, Johnston D (2006). Dopaminergic regulation of neuronal excitability through modulation of Ih in layer V entorhinal cortex. J Neurosci.

[44] Dyhrfjeld-Johnsen J, Morgan RJ, Soltesz I (2009). Double trouble? Potential for hyperexcitability following both channelopathic up- and downregulation of I(h) in epilepsy. Front Neurosci.

[45] George MS, Abbott LF, Siegelbaum SA (2009). HCN hyperpolarization-activated cation channels inhibit EPSPs by interactions with M-type K(+) channels. Nat Neurosci.

[46] Fan Y, Fricker D, Brager DH (2005). Activity-dependent decrease of excitability in rat hippocampal neurons through increases in I(h). Nat Neurosci.

[47] Narayanan R, Johnston D (2007). Long-term potentiation in rat hippocampal neurons is accompanied by spatially widespread changes in intrinsic oscillatory dynamics and excitability. Neuron.

[48] Pavlov I, Scimemi A, Savtchenko L, Kullmann DM, Walker MC (2011). I(h)-mediated depolarization enhances the temporal precision of neuronal integration. Nat Commun.

[49] Chen K, Aradi I, Thon N, Eghbal-Ahmadi M, Baram TZ, Soltesz I (2001). Persistently modified h-channels after complex febrile seizures convert the seizure-induced enhancement of inhibition to hyperexcitability. Nat Med.

[50] Breton JD, Stuart GJ (2009). Loss of sensory input increases the intrinsic excitability of layer 5 pyramidal neurons in rat barrel cortex. J Physiol.

[51] Kim CS, Chang PY, Johnston D (2012). Enhancement of dorsal hippocampal activity by knockdown of HCN1 channels leads to anxiolytic- and antidepressant-like behaviors. Neuron.

[52] Noam Y, Bernard C, Baram TZ (2011). Towards an integrated view of HCN channel role in epilepsy. Curr Opin Neurobiol.

[53] Lippert A, Booth V (2009). Understanding effects on excitability of simulated I (h) modulation in simple neuronal models. Biol Cybern.

[54] Mishra P, Narayanan R (2015). High-conductance states and A-type K+ channels are potential regulators of the conductance-current balance triggered by HCN channels. J Neurophysiol.

[55] Magee JC (1999). Dendritic lh normalizes temporal summation in hippocampal CA1 neurons. Nat Neurosci.

[56] Williams SR, Stuart GJ (2000). Site independence of EPSP time course is mediated by dendritic I(h) in neocortical pyramidal neurons. J Neurophysiol.

[57] Poolos N, Migliore M, Johnston D (2002). Pharmacological upregulation of h-channels reduces the excitability of pyramidal neuron dendrites. Nat Neurosci.

[58] Ashhad S, Narayanan R (2016). Active dendrites regulate the impact of gliotransmission on rat hippocampal pyramidal neurons. Proc Natl Acad Sci U S A.

[59] Ashhad S, Narayanan R (2019). Stores, channels, glue, and trees: active glial and active dendritic physiology. Mol Neurobiol.

[60] Kole MH, Brauer AU, Stuart GJ (2007). Inherited cortical HCN1 channel loss amplifies dendritic calcium electrogenesis and burst firing in a rat absence epilepsy model. J Physiol.

[61] Cole KS (1941). Rectification and inductance in the squid giant axon. J Gen Physiol.

[62] Cole KS, Baker RF (1941). Longitudinal impedance of the squid giant axon. J Gen Physiol.

[63] Hodgkin AL, Huxley AF (1952). A quantitative description of membrane current and its application to conduction and excitation in nerve. J Physiol.

[64] Mauro A (1961). Anomalous impedance, a phenomenological property of time-variant resistance. An analytic review. Biophys J.

[65] Cole KS (1932). Electrical phase angle of cell membranes. J Gen Physiol.

[66] Cole KS (1949). Some physical aspects of bioelectric phenomena. Proc Natl Acad Sci U S A.

[67] Cole KS (1968). Membranes, Ions and Impulses: A Chapter of Classical Biophysics.

[68] Mauro A, Conti F, Dodge F, Schor R (1970). Subthreshold behavior and phenomenological impedance of the squid giant axon. J Gen Physiol.

[69] Sabah NH, Leibovic KN (1969). Subthreshold oscillatory responses of the Hodgkin-Huxley cable model for the squid giant axon. Biophys J.

[70] Koch C (1984). Cable theory in neurons with active, linearized membranes. Biol Cybern.

[71] Smith TG, Wuerker RB, Frank K (1967). Membrane impedance changes during synaptic transmission in cat spinal motoneurons. J Neurophysiol.

[72] Rall W (1960). Membrane potential transients and membrane time constant of motoneurons. Exp Neurol.

[73] Gimbarzevsky B, Miura RM, Puil E (1984). Impedance profiles of peripheral and central neurons. Can J Physiol Pharmacol.

[74] Hutcheon B, Miura RM, Puil E (1996). Subthreshold membrane resonance in neocortical neurons. J Neurophysiol.

[75] Puil E, Gimbarzevsky B, Miura RM (1986). Quantification of membrane properties of trigeminal root ganglion neurons in guinea pigs. J Neurophysiol.

[76] Puil E, Meiri H, Yarom Y (1994). Resonant behavior and frequency preferences of thalamic neurons. J Neurophysiol.

[77] Schreiber S, Erchova I, Heinemann U, Herz AV (2004). Subthreshold resonance explains the frequency-dependent integration of periodic as well as random stimuli in the entorhinal cortex. J Neurophysiol.

[78] Hu H, Vervaeke K, Storm JF (2002). Two forms of electrical resonance at theta frequencies, generated by M-current, h-current and persistent Na+ current in rat hippocampal pyramidal cells. J Physiol.

[79] Gutfreund Y, Yarom Y, Segev I (1995). Subthreshold oscillations and resonant frequency in guinea-pig cortical neurons: physiology and modelling. J Physiol.

[80] Llinas RR (1988). The intrinsic electrophysiological properties of mammalian neurons: insights into central nervous system function. Science.

[81] Crawford AC, Fettiplace R (1981). An electrical tuning mechanism in turtle cochlear hair cells. J Physiol.

[82] Hutcheon B, Miura RM, Puil E (1996). Models of subthreshold membrane resonance in neocortical neurons. J Neurophysiol.

[83] Hutcheon B, Miura RM, Yarom Y, Puil E (1994). Low-threshold calcium current and resonance in thalamic neurons: a model of frequency preference. J Neurophysiol.

[84] Izhikevich EM (2007). Dynamical Systems in Neuroscience: The Geometry of Excitability and Bursting.

[85] Armstrong-Gold CE, Rieke F (2003). Bandpass filtering at the rod to second-order cell synapse in salamander (*Ambystoma tigrinum*) retina. J Neurosci.

[86] Demontis GC, Longoni B, Barcaro U, Cervetto L (1999). Properties and functional roles of hyperpolarization-gated currents in guinea-pig retinal rods. J Physiol.

[87] Erchova I, Kreck G, Heinemann U, Herz AV (2004). Dynamics of rat entorhinal cortex layer II and III cells: characteristics of membrane potential resonance at rest predict oscillation properties near threshold. J Physiol.

[88] Mao BQ, MacLeish PR, Victor JD (2003). Role of hyperpolarization-activated currents for the intrinsic dynamics of isolated retinal neurons. Biophys J.

[89] Mittal D, Narayanan R (2021). Resonating neurons stabilize heterogeneous grid-cell networks. Elife.

[90] Sinha M, Narayanan R (2015). HCN channels enhance spike phase coherence and regulate the phase of spikes and LFPs in the theta-frequency range. Proc Natl Acad Sci U S A.

[91] Sinha M, Narayanan R (2022). Active dendrites and local field potentials: biophysical mechanisms and computational explorations. Neuroscience.

[92] Ness TV, Remme MWH, Einevoll GT (2016). Active subthreshold dendritic conductances shape the local field potential. J Physiol-London.

[93] Ness TV, Remme MWH, Einevoll GT (2018). h-Type membrane current shapes the local field potential from populations of pyramidal neurons. J Neurosci.

[94] Hodgkin AL (1948). The local electric changes associated with repetitive action in a non-medullated axon. J Physiol.

[95] Ratte S, Hong S, De Schutter E, Prescott SA (2013). Impact of neuronal properties on network coding: roles of spike initiation dynamics and robust synchrony transfer. Neuron.

[96] Agueray Arcas B, Fairhall AL (2003). What causes a neuron to spike?. Neural Comput.

[97] Badel L, Gerstner W, Richardson MJ (2008). Spike-triggered averages for passive and resonant neurons receiving filtered excitatory and inhibitory synaptic drive. Phys Rev E Stat Nonlin Soft Matter Phys.

[98] Das A, Narayanan R (2014). Active dendrites regulate spectral selectivity in location-dependent spike initiation dynamics of hippocampal model neurons. J Neurosci.

[99] Das A, Narayanan R (2015). Active dendrites mediate stratified gamma-range coincidence detection in hippocampal model neurons. J Physiol.

[100] Das A, Narayanan R (2017). Theta-frequency selectivity in the somatic spike triggered average of rat hippocampal pyramidal neurons is dependent on HCN channels. J Neurophysiol.

[101] Ermentrout GB, Galan RF, Urban NN (2007). Relating neural dynamics to neural coding. Phys Rev Lett.

[102] Famulare M, Fairhall A (2010). Feature selection in simple neurons: how coding depends on spiking dynamics. Neural Comput.

[103] Hong S, Aguera y Arcas B, Fairhall AL (2007). Single neuron computation: from dynamical system to feature detector. Neural Comput.

[104] Prescott SA, De Koninck Y, Sejnowski TJ (2008). Biophysical basis for three distinct dynamical mechanisms of action potential initiation. PLoS Comput Biol.

[105] Prescott SA, Ratte S, De Koninck Y, Sejnowski TJ (2006). Nonlinear interaction between shunting and adaptation controls a switch between integration and coincidence detection in pyramidal neurons. J Neurosci.

[106] Prescott SA, Ratte S, De Koninck Y, Sejnowski TJ (2008). Pyramidal neurons switch from integrators in vitro to resonators under in vivo-like conditions. J Neurophysiol.

[107] Prescott SA, Sejnowski TJ (2008). Spike-rate coding and spike-time coding are affected oppositely by different adaptation mechanisms. J Neurosci.

[108] Mittal D, Narayanan R (2018). Degeneracy in the robust expression of spectral selectivity, subthreshold oscillations and intrinsic excitability of entorhinal stellate cells. J Neurophysiol.

[109] Abouzeid A, Ermentrout B (2009). Type-II phase resetting curve is optimal for stochastic synchrony. Phys Rev E Stat Nonlin Soft Matter Phys.

[110] Ermentrout B (1996). Type I membranes, phase resetting curves, and synchrony. Neural Comput.

[111] Golding NL, Oertel D (2012). Synaptic integration in dendrites: exceptional need for speed. J Physiol.

[112] Khurana S, Liu Z, Lewis AS, Rosa K, Chetkovich D, Golding NL (2012). An essential role for modulation of hyperpolarization-activated current in the development of binaural temporal precision. J Neurosci.

[113] Mathews PJ, Jercog PE, Rinzel J, Scott LL, Golding NL (2010). Control of submillisecond synaptic timing in binaural coincidence detectors by K(v)1 channels. Nat Neurosci.

[114] Das A, Rathour RK, Narayanan R (2017). Strings on a violin: location dependence of frequency tuning in active dendrites. Front Cell Neurosci.

[115] Jain A, Narayanan R (2020). Degeneracy in the emergence of spike-triggered average of hippocampal pyramidal neurons. Sci Rep.

[116] Ratte S, Lankarany M, Rho YA, Patterson A, Prescott SA (2014). Subthreshold membrane currents confer distinct tuning properties that enable neurons to encode the integral or derivative of their input. Front Cell Neurosci.

[117] Ratté S, Zhu Y, Lee KY, Prescott SA (2014). Criticality and degeneracy in injury-induced changes in primary afferent excitability and the implications for neuropathic pain. Elife.

[118] Angelo K, London M, Christensen SR, Hausser M (2007). Local and global effects of I(h) distribution in dendrites of mammalian neurons. J Neurosci.

[119] Khurana S, Remme MW, Rinzel J, Golding NL (2011). Dynamic interaction of Ih and IK-LVA during trains of synaptic potentials in principal neurons of the medial superior olive. J Neurosci.

[120] Lorincz A, Notomi T, Tamas G, Shigemoto R, Nusser Z (2002). Polarized and compartment-dependent distribution of HCN1 in pyramidal cell dendrites. Nat Neurosci.

[121] Nusser Z (2009). Variability in the subcellular distribution of ion channels increases neuronal diversity. Trends Neurosci.

[122] Nusser Z (2012). Differential subcellular distribution of ion channels and the diversity of neuronal function. Curr Opin Neurobiol.

[123] Johnston D, Narayanan R (2008). Active dendrites: colorful wings of the mysterious butterflies. Trends Neurosci.

[124] Roth FC, Hu H (2020). An axon-specific expression of HCN channels catalyzes fast action potential signaling in GABAergic interneurons. Nat Commun.

[125] Ko KW, Rasband MN, Meseguer V, Kramer RH, Golding NL (2016). Serotonin modulates spike probability in the axon initial segment through HCN channels. Nat Neurosci.

[126] Huang Z, Lujan R, Kadurin I (2011). Presynaptic HCN1 channels regulate Cav3.2 activity and neurotransmission at select cortical synapses. Nat Neurosci.

[127] Kole MH, Hallermann S, Stuart GJ (2006). Single Ih channels in pyramidal neuron dendrites: properties, distribution, and impact on action potential output. J Neurosci.

[128] Kalmbach BE, Chitwood RA, Dembrow NC, Johnston D (2013). Dendritic generation of mGluR-mediated slow afterdepolarization in layer 5 neurons of prefrontal cortex. J Neurosci.

[129] Magee JC (2000). Dendritic integration of excitatory synaptic input. Nat Rev Neurosci.

[130] Rall W (1967). Distinguishing theoretical synaptic potentials computed for different soma-dendritic distributions of synaptic input. J Neurophysiol.

[131] Rall W, Kandel ER (1977). Handbook of Physiology. The Nervous System. Cellular Biology of Neurons. Vol 1.

[132] Rall W, Burke RE, Smith TG, Nelson PG, Frank K (1967). Dendritic location of synapses and possible mechanisms for the monosynaptic EPSP in motoneurons. J Neurophysiol.

[133] Narayanan R, Johnston D (2012). Functional maps within a single neuron. J Neurophysiol.

[134] Hu H, Vervaeke K, Graham LJ, Storm JF (2009). Complementary theta resonance filtering by two spatially segregated mechanisms in CA1 hippocampal pyramidal neurons. J Neurosci.

[135] Vaidya SP, Johnston D (2013). Temporal synchrony and gamma-to-theta power conversion in the dendrites of CA1 pyramidal neurons. Nat Neurosci.

[136] Dhupia N, Rathour RK, Narayanan R (2015). Dendritic atrophy constricts functional maps in resonance and impedance properties of hippocampal model neurons. Front Cell Neurosci.

[137] Rathour RK, Narayanan R (2012). Influence fields: a quantitative framework for representation and analysis of active dendrites. J Neurophysiol.

[138] Bieri KW, Bobbitt KN, Colgin LL (2014). Slow and fast gamma rhythms coordinate different spatial coding modes in hippocampal place cells. Neuron.

[139] Colgin LL, Denninger T, Fyhn M (2009). Frequency of gamma oscillations routes flow of information in the hippocampus. Nature.

[140] Colgin LL, Moser EI (2010). Gamma oscillations in the hippocampus. Phys Ther.

[141] Fernandez-Ruiz A, Oliva A, Nagy GA, Maurer AP, Berenyi A, Buzsaki G (2017). Entorhinal-CA3 dual-input control of spike timing in the hippocampus by theta-gamma coupling. Neuron.

[142] Alon U (2019). An Introduction to Systems Biology: Design Principles of Biological Circuits.

[143] Haas JS, Dorval AD, White JA (2007). Contributions of Ih to feature selectivity in layer II stellate cells of the entorhinal cortex. J Comput Neurosci.

[144] Haas JS, White JA (2002). Frequency selectivity of layer II stellate cells in the medial entorhinal cortex. J Neurophysiol.

[145] White JA, Budde T, Kay AR (1995). A bifurcation analysis of neuronal subthreshold oscillations. Biophys J.

[146] White JA, Klink R, Alonso A, Kay AR (1998). Noise from voltage-gated ion channels may influence neuronal dynamics in the entorhinal cortex. J Neurophysiol.

[147] Mittal D, Narayanan R (2022). Heterogeneous stochastic bifurcations explain intrinsic oscillatory patterns in entorhinal cortical stellate cells. Proc Natl Acad Sci U S A.

[148] Fransen E, Alonso AA, Dickson CT, Magistretti J, Hasselmo ME (2004). Ionic mechanisms in the generation of subthreshold oscillations and action potential clustering in entorhinal layer II stellate neurons. Hippocampus.

[149] Yoshida M, Giocomo LM, Boardman I, Hasselmo ME (2011). Frequency of subthreshold oscillations at different membrane potential voltages in neurons at different anatomical positions on the dorsoventral axis in the rat medial entorhinal cortex. J Neurosci.

[150] Accili EA, Proenza C, Baruscotti M, DiFrancesco D (2002). From funny current to HCN channels: 20 years of excitation. News Physiol Sci.

[151] Kaupp UB, Seifert R (2001). Molecular diversity of pacemaker ion channels. Annu Rev Physiol.

[152] Luthi A, McCormick DA (1998). H-current: properties of a neuronal and network pacemaker. Neuron.

[153] Klink R, Alonso A (1993). Ionic mechanisms for the subthreshold oscillations and differential electroresponsiveness of medial entorhinal cortex layer II neurons. J Neurophysiol.

[154] Varga V, Hangya B, Kranitz K (2008). The presence of pacemaker HCN channels identifies theta rhythmic GABAergic neurons in the medial septum. J Physiol.

[155] Alonso A, Llinas RR (1989). Subthreshold Na^+^-dependent theta-like rhythmicity in stellate cells of entorhinal cortex layer II. Nature.

[156] Hoffman DA, Magee JC, Colbert CM, Johnston D (1997). K+ channel regulation of signal propagation in dendrites of hippocampal pyramidal neurons. Nature.

[157] Kim J, Wei DS, Hoffman DA (2005). Kv4 potassium channel subunits control action potential repolarization and frequency-dependent broadening in rat hippocampal CA1 pyramidal neurones. J Physiol.

[158] Rathour RK, Malik R, Narayanan R (2016). Transient potassium channels augment degeneracy in hippocampal active dendritic spectral tuning. Sci Rep.

[159] Frick A, Magee J, Johnston D (2004). LTP is accompanied by an enhanced local excitability of pyramidal neuron dendrites. Nat Neurosci.

[160] Migliore M, Hoffman DA, Magee JC, Johnston D (1999). Role of an A-type K+ conductance in the back-propagation of action potentials in the dendrites of hippocampal pyramidal neurons. J Comput Neurosci.

[161] Chen X, Yuan LL, Zhao C (2006). Deletion of Kv4.2 gene eliminates dendritic A-type K+ current and enhances induction of long-term potentiation in hippocampal CA1 pyramidal neurons. J Neurosci.

[162] Rathour RK, Narayanan R (2019). Degeneracy in hippocampal physiology and plasticity. Hippocampus.

[163] Goaillard JM, Marder E (2021). Ion channel degeneracy, variability, and covariation in neuron and circuit resilience. Annu Rev Neurosci.

[164] Mishra P, Narayanan R (2021). Ion-channel regulation of response decorrelation in a heterogeneous multi-scale model of the dentate gyrus. Curr Res Neurobiol.

[165] Mishra P, Narayanan R (2021). Ion-channel degeneracy: multiple ion channels heterogeneously regulate intrinsic physiology of rat hippocampal granule cells. Physiol Rep.

[166] Goldman MS, Golowasch J, Marder E, Abbott LF (2001). Global structure, robustness, and modulation of neuronal models. J Neurosci.

[167] Rathour RK, Narayanan R (2012). Inactivating ion channels augment robustness of subthreshold intrinsic response dynamics to parametric variability in hippocampal model neurons. J Physiol.

[168] Rathour RK, Narayanan R (2014). Homeostasis of functional maps in active dendrites emerges in the absence of individual channelostasis. Proc Natl Acad Sci U S A.

[169] Mishra P, Narayanan R (2020). Heterogeneities in intrinsic excitability and frequency-dependent response properties of granule cells across the blades of the rat dentate gyrus. J Neurophysiol.

[170] Kispersky TJ, Caplan JS, Marder E (2012). Increase in sodium conductance decreases firing rate and gain in model neurons. J Neurosci.

[171] Drion G, O’Leary T, Marder E (2015). Ion channel degeneracy enables robust and tunable neuronal firing rates. Proc Natl Acad Sci U S A.

[172] Mukunda CL, Narayanan R (2017). Degeneracy in the regulation of short-term plasticity and synaptic filtering by presynaptic mechanisms. J Physiol.

[173] Narayanan R, Dougherty KJ, Johnston D (2010). Calcium store depletion induces persistent perisomatic increases in the functional density of h channels in hippocampal pyramidal neurons. Neuron.

[174] Josselyn SA, Frankland PW (2018). Memory allocation: mechanisms and function. Annu Rev Neurosci.

[175] Josselyn SA, Tonegawa S (2020). Memory engrams: recalling the past and imagining the future. Science.

[176] Kol A, Goshen I (2020). The memory orchestra: the role of astrocytes and oligodendrocytes in parallel to neurons. Curr Opin Neurobiol.

[177] Kim SJ, Linden DJ (2007). Ubiquitous plasticity and memory storage. Neuron.

[178] Mishra P, Narayanan R (2021). Stable continual learning through structured multiscale plasticity manifolds. Curr Opin Neurobiol.

[179] Lisman J, Cooper K, Sehgal M, Silva AJ (2018). Memory formation depends on both synapse-specific modifications of synaptic strength and cell-specific increases in excitability. Nat Neurosci.

[180] Titley HK, Brunel N, Hansel C (2017). Toward a neurocentric view of learning. Neuron.

[181] Nelson SB, Turrigiano GG (2008). Strength through diversity. Neuron.

[182] Frick A, Johnston D (2005). Plasticity of dendritic excitability. J Neurobiol.

[183] Shah MM, Hammond RS, Hoffman DA (2010). Dendritic ion channel trafficking and plasticity. Trends Neurosci.

[184] Wang Z, Xu NL, Wu CP, Duan S, Poo MM (2003). Bidirectional changes in spatial dendritic integration accompanying long-term synaptic modifications. Neuron.

[185] Mishra P, Narayanan R (2022). Conjunctive changes in multiple ion channels mediate activity-dependent intrinsic plasticity in hippocampal granule cells. iScience.

[186] Clemens AM, Johnston D (2014). Age- and location-dependent differences in store depletion-induced h-channel plasticity in hippocampal pyramidal neurons. J Neurophysiol.

[187] Ashhad S, Johnston D, Narayanan R (2015). Activation of InsP3 receptors is sufficient for inducing graded intrinsic plasticity in rat hippocampal pyramidal neurons. J Neurophysiol.

[188] Brager DH, Johnston D (2007). Plasticity of intrinsic excitability during long-term depression is mediated through mGluR-dependent changes in I(h) in hippocampal CA1 pyramidal neurons. J Neurosci.

[189] Poolos NP, Bullis JB, Roth MK (2006). Modulation of h-channels in hippocampal pyramidal neurons by p38 mitogen-activated protein kinase. J Neurosci.

[190] Shin M, Chetkovich DM (2007). Activity-dependent regulation of h channel distribution in hippocampal CA1 pyramidal neurons. J Biol Chem.

[191] Wang M, Ramos BP, Paspalas CD (2007). Alpha2A-adrenoceptors strengthen working memory networks by inhibiting cAMP-HCN channel signaling in prefrontal cortex. Cell.

[192] DiFrancesco D, Mangoni M (1994). Modulation of single hyperpolarization-activated channels (i(f)) by cAMP in the rabbit sino-atrial node. J Physiol.

[193] Rosenkranz JA, Johnston D (2007). State-dependent modulation of amygdala inputs by dopamine-induced enhancement of sodium currents in layer V entorhinal cortex. J Neurosci.

[194] Dembrow N, Johnston D (2014). Subcircuit-specific neuromodulation in the prefrontal cortex. Front Neural Circuits.

[195] Dembrow NC, Chitwood RA, Johnston D (2010). Projection-specific neuro-modulation of medial prefrontal cortex neurons. J Neurosci.

[196] van Welie I, van Hooft JA, Wadman WJ (2004). Homeostatic scaling of neuronal excitability by synaptic modulation of somatic hyperpolarization-activated Ih channels. Proc Natl Acad Sci U S A.

[197] Honnuraiah S, Narayanan R (2013). A calcium-dependent plasticity rule for HCN channels maintains activity homeostasis and stable synaptic learning. PLoS One.

[198] Poolos NP, Johnston D (2012). Dendritic ion channelopathy in acquired epilepsy. Epilepsia.

[199] Brager DH, Akhavan AR, Johnston D (2012). Impaired dendritic expression and plasticity of h-channels in the fmr1(−/y) mouse model of fragile X syndrome. Cell Rep.

[200] Jung S, Bullis JB, Lau IH, Jones TD, Warner LN, Poolos NP (2010). Downregulation of dendritic HCN channel gating in epilepsy is mediated by altered phosphorylation signaling. J Neurosci.

[201] Jung S, Jones TD, Lugo JN (2007). Progressive dendritic HCN channelopathy during epileptogenesis in the rat pilocarpine model of epilepsy. J Neurosci.

[202] Jung S, Warner LN, Pitsch J, Becker AJ, Poolos NP (2011). Rapid loss of dendritic HCN channel expression in hippocampal pyramidal neurons following status epilepticus. J Neurosci.

[203] Shah MM (2012). HCN1 channels: a new therapeutic target for depressive disorders?. Sci Signal.

[204] Shah MM, Anderson AE, Leung V, Lin X, Johnston D (2004). Seizure-induced plasticity of h channels in entorhinal cortical layer III pyramidal neurons. Neuron.

[205] Narayanan R, Johnston D (2010). The h current is a candidate mechanism for regulating the sliding modification threshold in a BCM-like synaptic learning rule. J Neurophysiol.

[206] Bienenstock EL, Cooper LN, Munro PW (1982). Theory for the development of neuron selectivity: orientation specificity and binocular interaction in visual cortex. J Neurosci.

[207] Shouval HZ, Bear MF, Cooper LN (2002). A unified model of NMDA receptor-dependent bidirectional synaptic plasticity. Proc Natl Acad Sci U S A.

[208] Cooper LN, Bear MF (2012). The BCM theory of synapse modification at 30: interaction of theory with experiment. Nat Rev Neurosci.

[209] Anirudhan A, Narayanan R (2015). Analogous synaptic plasticity profiles emerge from disparate channel combinations. J Neurosci.

[210] Bear MF (1995). Mechanism for a sliding synaptic modification threshold. Neuron.

[211] Castellani GC, Quinlan EM, Cooper LN, Shouval HZ (2001). A biophysical model of bidirectional synaptic plasticity: dependence on AMPA and NMDA receptors. Proc Natl Acad Sci U S A.

[212] Magee JC, Johnston D (1997). A synaptically controlled, associative signal for Hebbian plasticity in hippocampal neurons. Science.

[213] Losonczy A, Makara JK, Magee JC (2008). Compartmentalized dendritic plasticity and input feature storage in neurons. Nature.

[214] Lin MT, Lujan R, Watanabe M, Adelman JP, Maylie J (2008). SK2 channel plasticity contributes to LTP at Schaffer collateral-CA1 synapses. Nat Neurosci.

[215] Nagaraj H, Narayanan R (2023). Plasticity manifolds and degeneracy govern circadian oscillations of neuronal intrinsic properties in the suprachiasmatic nucleus. iScience.

[216] Shridhar S, Mishra P, Narayanan R (2022). Dominant role of adult neurogenesis-induced structural heterogeneities in driving plasticity heterogeneity in dentate gyrus granule cells. Hippocampus.

[217] Ashhad S, Narayanan R (2013). Quantitative interactions between the A-type K^+^ current and inositol trisphosphate receptors regulate intraneuronal Ca^2+^ waves and synaptic plasticity. J Physiol.

[218] Baruscotti M, Bucchi A, Difrancesco D (2005). Physiology and pharmacology of the cardiac pacemaker (“funny”) current. Pharmacol Ther.

[219] Cichon J, Wasilczuk AZ, Looger LL, Contreras D, Kelz MB, Proekt A (2023). Ketamine triggers a switch in excitatory neuronal activity across neocortex. Nat Neurosci.

[220] Higuchi H, Funahashi M, Miyawaki T (2003). Suppression of the hyperpolarization-activated inward current contributes to the inhibitory actions of propofol on rat CA1 and CA3 pyramidal neurons. Neurosci Res.

[221] Meng QT, Xia ZY, Liu J, Bayliss DA, Chen X (2011). Local anesthetic inhibits hyperpolarization-activated cationic currents. Mol Pharmacol.

[222] Sirois JE, Lynch C, Bayliss DA (2002). Convergent and reciprocal modulation of a leak K^+^ current and I(h) by an inhalational anaesthetic and neurotransmitters in rat brainstem motoneurones. J Physiol.

[223] Chen X, Sirois JE, Lei Q, Talley EM, Lynch C, Bayliss DA (2005). HCN subunit-specific and cAMP-modulated effects of anesthetics on neuronal pacemaker currents. J Neurosci.

[224] Chen X, Shu S, Bayliss DA (2009). HCN1 channel subunits are a molecular substrate for hypnotic actions of ketamine. J Neurosci.

[225] Chen X, Shu S, Kennedy DP, Willcox SC, Bayliss DA (2009). Subunit-specific effects of isoflurane on neuronal Ih in HCN1 knockout mice. J Neurophysiol.

[226] Kim CS, Brager DH, Johnston D (2017). Perisomatic changes in h-channels regulate depressive behaviors following chronic unpredictable stress. Mol Psychiatry.

[227] Kim J, Lei Y, Lu XY, Kim CS (2022). Glucocorticoid-glucocorticoid receptor-HCN1 channels reduce neuronal excitability in dorsal hippocampal CA1 neurons. Mol Psychiatry.

[228] Fisher DW, Han Y, Lyman KA (2018). HCN channels in the hippocampus regulate active coping behavior. J Neurochem.

[229] Zhong P, Vickstrom CR, Liu X (2018). HCN2 channels in the ventral tegmental area regulate behavioral responses to chronic stress. Elife.

[230] Stegen M, Kirchheim F, Hanuschkin A, Staszewski O, Veh RW, Wolfart J (2012). Adaptive intrinsic plasticity in human dentate gyrus granule cells during temporal lobe epilepsy. Cereb Cortex.

[231] Arnold EC, McMurray C, Gray R, Johnston D (2019). Epilepsy-induced reduction in HCN channel expression contributes to an increased excitability in dorsal, but not ventral, hippocampal CA1 neurons. eNeuro.

[232] Bernard C, Anderson A, Becker A, Poolos NP, Beck H, Johnston D (2004). Acquired dendritic channelopathy in temporal lobe epilepsy. Science.

[233] Lerche H, Shah M, Beck H, Noebels J, Johnston D, Vincent A (2013). Ion channels in genetic and acquired forms of epilepsy. J Physiol.

[234] Shin M, Brager D, Jaramillo TC, Johnston D, Chetkovich DM (2008). Mislocalization of h channel subunits underlies h channelopathy in temporal lobe epilepsy. Neurobiol Dis.

[235] Stober TM, Batulin D, Triesch J, Narayanan R, Jedlicka P (2023). Degeneracy in epilepsy: multiple routes to hyperexcitable brain circuits and their repair. Commun Biol.

[236] Kazmierska-Grebowska P, Jankowski MM, MacIver MB (2023). Missing puzzle pieces in dementia research: HCN channels and theta oscillations. Aging Dis.

[237] Saito Y, Inoue T, Zhu G (2012). Hyperpolarization-activated cyclic nucleotide gated channels: a potential molecular link between epileptic seizures and Abeta generation in Alzheimer’s disease. Mol Neurodegener.

[238] Brager DH, Johnston D (2014). Channelopathies and dendritic dysfunction in fragile X syndrome. Brain Res Bull.

[239] Brandalise F, Kalmbach BE, Mehta P (2020). Fragile X mental retardation protein bidirectionally controls dendritic Ih in a cell type-specific manner between mouse hippocampus and prefrontal cortex. J Neurosci.

[240] Deng PY, Klyachko VA (2021). Channelopathies in fragile X syndrome. Nat Rev Neurosci.

[241] Kalmbach BE, Johnston D, Brager DH (2015). Cell-type specific channelopathies in the prefrontal cortex of the fmr1−/y mouse model of fragile X syndrome(1,2,3). eNeuro.

[242] Graves AR, Roth RH, Tan HL (2021). Visualizing synaptic plasticity in vivo by large-scale imaging of endogenous AMPA receptors. Elife.

[243] Roth RH, Cudmore RH, Tan HL, Hong I, Zhang Y, Huganir RL (2020). Cortical synaptic AMPA receptor plasticity during motor learning. Neuron.

[244] Zhang Y, Cudmore RH, Lin DT, Linden DJ, Huganir RL (2015). Visualization of NMDA receptor-dependent AMPA receptor synaptic plasticity in vivo. Nat Neurosci.

[245] Choquet D, Sainlos M, Sibarita JB (2021). Advanced imaging and labelling methods to decipher brain cell organization and function. Nat Rev Neurosci.

[246] Kalmbach BE, Buchin A, Long B (2018). h-Channels contribute to divergent intrinsic membrane properties of supragranular pyramidal neurons in human versus mouse cerebral cortex. Neuron.

[247] Beaulieu-Laroche L, Brown NJ, Hansen M (2021). Allometric rules for mammalian cortical layer 5 neuron biophysics. Nature.

[248] Vyas S, Golub MD, Sussillo D, Shenoy KV (2020). Computation through neural population dynamics. Annu Rev Neurosci.

[249] Langdon C, Genkin M, Engel TA (2023). A unifying perspective on neural manifolds and circuits for cognition. Nat Rev Neurosci.

[250] Gurnani H, Cayco Gajic NA (2023). Signatures of task learning in neural representations. Curr Opin Neurobiol.

[251] Pereira TD, Shaevitz JW, Murthy M (2020). Quantifying behavior to understand the brain. Nat Neurosci.

[252] Kaplan HS, Zimmer M (2020). Brain-wide representations of ongoing behavior: a universal principle?. Curr Opin Neurobiol.

[253] Lin A, Witvliet D, Hernandez-Nunez L, Linderman SW, Samuel ADT, Venkatachalam V (2022). Imaging whole-brain activity to understand behavior. Nat Rev Phys.

[254] Westlin C, Theriault JE, Katsumi Y (2023). Improving the study of brain-behavior relationships by revisiting basic assumptions. Trends Cogn Sci.

[255] Turrigiano G (2011). Too many cooks? Intrinsic and synaptic homeostatic mechanisms in cortical circuit refinement. Annu Rev Neurosci.

[256] Biel M, Wahl-Schott C, Michalakis S, Zong X (2009). Hyperpolarization-activated cation channels: from genes to function. Physiol Rev.

